# Mitochondrial‐Targeted CS@KET/P780 Nanoplatform for Site‐Specific Delivery and High‐Efficiency Cancer Immunotherapy in Hepatocellular Carcinoma

**DOI:** 10.1002/advs.202308027

**Published:** 2024-02-02

**Authors:** Shanshan Liu, Hailong Tian, Hui Ming, Tingting Zhang, Yajie Gao, Ruolan Liu, Lihua Chen, Chen Yang, Edouard C. Nice, Canhua Huang, Jinku Bao, Wei Gao, Zheng Shi

**Affiliations:** ^1^ Clinical Medical College Affiliated Hospital of Chengdu University Chengdu University Chengdu 610106 China; ^2^ Department of Clinical Pharmacy School of Pharmacy Zunyi Medical University Zunyi 563006 China; ^3^ State Key Laboratory of Biotherapy and Cancer Center West China Hospital and West China School of Basic Medical Sciences & Forensic Medicine Sichuan University Collaborative Innovation Center for Biotherapy Chengdu 610041 China; ^4^ The First Affiliated Hospital of Ningbo University Ningbo 315020 China; ^5^ School of Basic Medical Sciences Chengdu University of Traditional Chinese Medicine Chengdu 611137 China; ^6^ Department of Biochemistry and Molecular Biology Monash University Clayton VIC 3800 Australia; ^7^ College of Life Sciences Sichuan University Chengdu 610064 China; ^8^ Clinical Genetics Laboratory Affiliated Hospital & Clinical Medical College of Chengdu University Chengdu 610081 China

**Keywords:** chemophototherapy, hepatocellular carcinoma, immunotherapy, ketoconazole, mitochondrial targeted

## Abstract

Hepatocellular carcinoma (HCC) is a form of malignancy with limited curative options available. To improve therapeutic outcomes, it is imperative to develop novel, potent therapeutic modalities. Ketoconazole (KET) has shown excellent therapeutic efficacy against HCC by eliciting apoptosis. However, its limited water solubility hampers its application in clinical treatment. Herein, a mitochondria‐targeted chemo‐photodynamic nanoplatform, CS@KET/P780 NPs, is designed using a nanoprecipitation strategy by integrating a newly synthesized mitochondria‐targeted photosensitizer (P780) and chemotherapeutic agent KET coated with chondroitin sulfate (CS) to amplify HCC therapy. In this nanoplatform, CS confers tumor‐targeted and subsequently pH‐responsive drug delivery behavior by binding to glycoprotein CD44, leading to the release of P780 and KET. Mechanistically, following laser irradiation, P780 targets and destroys mitochondrial integrity, thus inducing apoptosis through the enhancement of reactive oxygen species (ROS) buildup. Meanwhile, KET‐induced apoptosis synergistically enhances the anticancer effect of P780. In addition, tumor cells undergoing apoptosis can trigger immunogenic cell death (ICD) and a longer‐term antitumor response by releasing tumor‐associated antigens (TAAs) and damage‐associated molecular patterns (DAMPs), which together contribute to improved therapeutic outcomes in HCC. Taken together, CS@KET/P780 NPs improve the bioavailability of KET and exhibit excellent therapeutic efficacy against HCC by exerting chemophototherapy and antitumor immunity.

## Graphical abstract

A mitochondria‐targeted chemophotodynamic nanoplatform, chondroitin sulfate (CS)@ketoconazole (KET)/P780 nanoplatforms (NPs), is designed using a nanoprecipitation strategy by integrating a newly synthesized mitochondria‐targeted photosensitizer (P780) and chemotherapeutic agent ketoconazole coated with chondroitin sulfate to amplify hepatocellular carcinoma therapy.

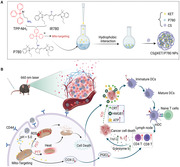

## 1 Introduction

Hepatocellular carcinoma (HCC) is a prevalent form of primary liver cancer and ranks as the second most significant contributor to cancer‐related mortality on a global scale.^[^ ^1^ ^]^ Surgical resection remains the main treatment for HCC, with the best long‐term survival observed for early‐stage HCC. However, up to 70% of patients inevitably recur, and treatment for HCC is still far from optimal.^[^ ^2^ ^]^ Therefore, the creation of innovative treatment approaches is urgently required to enhance therapeutic results.

As opposed to traditional medication development, drug repurposing offers a time‐ and money‐saving method for drug discovery by exploring new uses for existing pharmaceuticals.^[^ ^3^ ^]^ Ketoconazole (KET) is a derivative of imidazole that functions as a broad‐spectrum antifungal.^[^ ^4^ ^]^ There is growing evidence suggesting KET possesses considerable antitumor properties for several forms of cancer.^[^ ^5^ ^]^ Furthermore, oral administration of ketoconazole has been proven to be relatively safe in cancer patients with no obvious increased toxicity.^[^ ^6^ ^]^ Moreover, our previous studies have demonstrated KET downregulates cyclooxygenase‐2 (COX‐2) in hepatocellular carcinoma, increasing mitophagy and resulting in apoptosis.^[^ ^7^ ^]^ However, poor bioavailability and water solubility restrict its practical use. Recently, it was shown that nanodrug delivery may successfully increase the water solubility and bioavailability of insoluble pharmaceuticals, demonstrating its promise.^[^ ^8^ ^]^

For solid tumors, combining chemotherapy with phototherapy (photothermal therapy and photodynamic therapy) looks to be a promising therapeutic plan.^[^ ^9^ ^]^ Photodynamic therapy (PDT) elicits potent anticancer properties by triggering apoptosis in cancerous cells through the augmentation of fatal reactive oxygen species (ROS) levels under the influence of near‐infrared (NIR) laser irradiation.^[^ ^10^ ^]^ In addition, chemotherapy can amplify the therapeutic efficiency of phototherapy, offsetting the short half‐life of photosensitizers (PSs). Studies have demonstrated that phototherapy can induce apoptosis, leading to immunogenic cell death (ICD), in which dying cells release tumor‐associated antigens and damage‐associated molecular patterns (DAMPs), such as calreticulin (CRT), adenosine triphosphate (ATP) and high‐mobility group box‐1 (HMGB1).^[^ ^11^ ^]^ This progress can enhance the immunogenicity of the tumor microenvironment and improve the effectiveness of therapeutic interventions.^[^ ^12^ ^]^ Notably, the targeting delivery of PSs to crucial cellular positions, such as specific organelles, can significantly augment the therapeutic efficacy against cancer.^[^ ^13^ ^]^ Mitochondria play a pivotal role in regulating cellular growth, metabolism, apoptosis, and other essential processes.^[^ ^14^ ^]^ Moreover, the majority of intracellular ROS are generated within mitochondria, conferring significant adaptability for tumor survival in hostile microenvironments.^[^ ^15^ ^]^ Consequently, it is justifiable to consider mitochondria as targeted subcellular organelles for drug delivery and therapy.

**Scheme 1:**
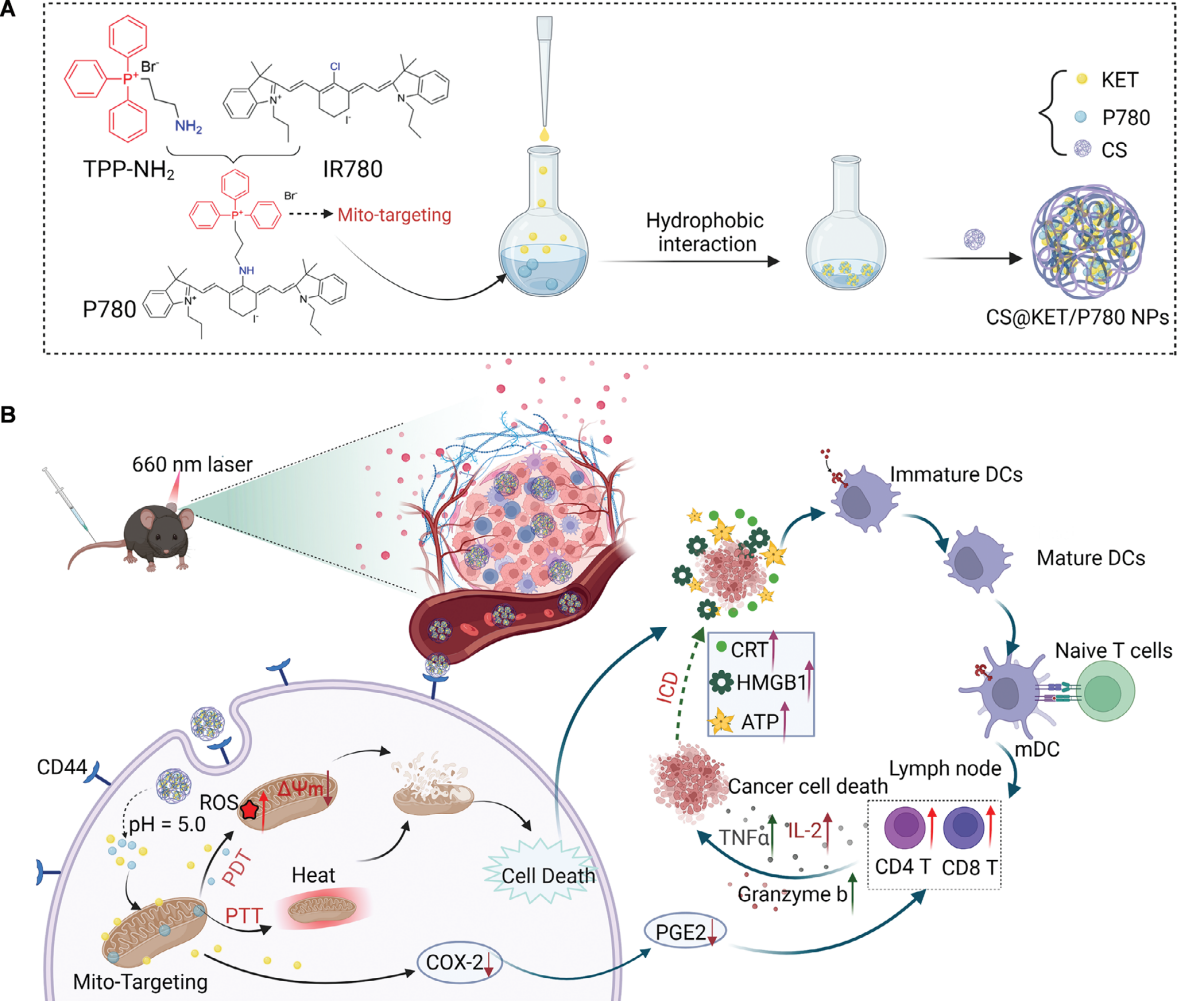
A) The chemical structure and generation of CS@KET/P780 NPs. B) Schematic showing CS@KET/P780 NPs executing apoptosis and anti‐tumor immunity during hepatoma therapy. Created with BioRender.com.

Herein, we have designed a mitochondria‐targeted chemo‐phototherapy nanoplatform (CS@KET/P780 NPs) consisting of KET, P780, and CS. Specifically, mitochondria‐targeting photosensitizer (P780) was synthesized by introducing mitochondrial targeting molecular modification (TPP) onto IR780. Then, P780 was co‐assembled with the chemotherapy drug KET to form KET/P780 nanoparticles (KET/P780 NPs). To mitigate potential circulating toxicity, KET/P780 NPs were further coated with tumor‐targeting CS to yield the CS@KET/P780 nanoplatform (CS@KET/P780 NPs, **Scheme** **1**A). In this nanoplatform, CS confers tumor‐targeting and subsequently pH‐responsive drug delivery behavior by binding to glycoprotein CD44, thus releasing P780 and KET. After dissociation, P780 can build up in mitochondria and destroy mitochondrial integrity through photothermal and photodynamic effects, thus leading to the induction of apoptosis via increasing ROS accumulation. Meanwhile, KET‐induced apoptosis can synergistically amplify the anticancer effect of P780. In addition, tumor cells undergoing apoptosis can induce ICD and a longer‐term antitumor response by releasing tumor‐associated antigens (TAAs) and DAMPs, which collectively enhance the treatment outcomes in an HCC mouse model (Scheme 1B). In summary, this novel nanoplatform improved the bioavailability of KET and exhibited excellent therapeutic efficacy against HCC by exerting PTT/PDT, chemotherapy, and antitumor immunity, suggesting an alternative strategy for effective treatment of HCC.

## 2 Results and Discussion

### 2.1 Synthesis and Characterization of CS@KET/P780 NPs

In the first step, the synthesis routes for TPP‐NH_2_ are displayed in Figure S1A, Supporting Information. The effective synthesis of TPP‐NH_2_ was confirmed through the use of ^1^H NMR and HR‐MS (high resolution mass spectrometry) (Figure S1B,C, Supporting Information). In the second step, the synthesis routes for P780 are presented in Figure S2A, Supporting Information. As shown in Figure S2B–D, Supporting Information the results of ^1^H NMR, ^13^C NMR, and HR‐MS verified the successful synthesis of P780 molecules.

**Figure 1:**
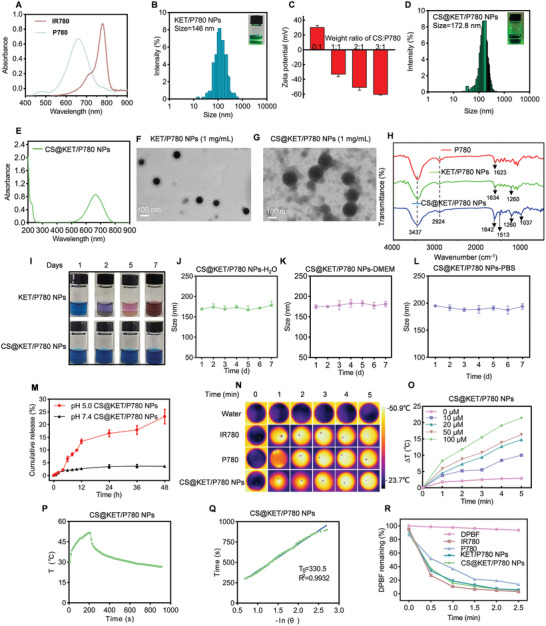
Synthesis and characterization of CS@KET/P780 NPs. A) UV−vis spectra of IR780 and P780 in carbinol. B) Tyndall effect and size distribution of KET/P780 NPs. C) Zeta potentials of CS@KET/P780 NPs at different weight ratios of CS and P780. D) Tyndall effect and size distribution of CS@KET/P780 NPs. E) UV−vis spectra of CS@KET/P780 NPs in water. F,G) TEM image of KET/P780 NPs and CS@KET/P780 NPs. H) FTIR spectra of P780, KET/P780 NPs and CS@KET/P780 NPs. I) Photographs of KET/P780 NPs and CS@KET/P780 NPs at indicated time points. J) Size change of CS@KET/P780 NPs in H_2_O solution over a week (*n* = 3). K,L) Size change of CS@KET/P780 NPs in K) DMEM and L) PBS solution over a week (*n* = 3). M) Release profiles of P780 at pH 5.0 and 7.4. N) Infrared thermal images of water, IR780, P780 and CS@KET/P780 NPs after NIR laser irradiation (equivalent IR780/P780 concentration of 100 × 10^−6^ m, 660 nm for CS@KET/P780 NPs and P780, 808 nm for IR780, 1.0 W cm^−2^, 5 min). O) Temperature variations caused by 660 nm laser irradiation (1.0 W cm^−2^, 5 min) at various concentrations of CS@KET/P780 NPs. P) Temperature profile of CS@KET/P780 NPs in water after 200 s irradiation with a laser (*λ* = 660 nm) at a power of 1.0 W cm^−2^. Q) Linear time data versus ‐ln(*θ*) obtained from the cooling period of CS@KET/P780 NPs. R) Residual levels of DPBF in IR780, P780, KET/P780 NPs, CS@KET/P780 NPs (equivalent IR780/P780 concentration of 2.5 × 10^−6^ m) after NIR laser irradiation (808 nm for IR780, 660 nm for P780, KET/P780 NPs, and CS@KET/P780 NPs). The error bars represent the means ± SD (*n* = 3).

Furthermore, the shift in UV–vis absorbance spectra of P780 to 660 nm compared with IR780 (808 nm) further indicated the successful synthesis of P780 (**Figure** **1**A). KET/P780 NPs were prepared by the nanoprecipitation method described in Scheme 1A. Based on their molecular structure, hydrophobic and π−π interactions are responsible for the co‐assembly of KET and P780 nanoparticles. In order to enhance the efficiency of the nanoplatform, the dispersion ability was evaluated by dynamic light scattering (DLS) at different mass ratios of KET and P780. Superior dispersion and particle size were observed at a mass ratio of 1:1 (Figure 1B and Figure S3A–D, Supporting Information). Additionally, we also assessed the zeta potential of CS@KET/P780 NPs at different mass ratios of CS and P780 (Figure 1C). It has been reported that nanoparticles with approximately −30 mV zeta potential exhibit solution stability because of strong electrostatic interactions.^[^ ^16^ ^]^ Therefore, CS@KET/P780 NPs at a mass ratio 1:1:1 (CS/KET/P780) were chosen as the final formulation for further study.

The Tyndall effect and DLS confirmed that the CS@KET/P780 NPs have an appropriate mean particle size (172.8 nm) (Figure 1D). The UV–vis spectra at 660 nm confirmed the successful preparation of CS@KET/P780 NPs (Figure 1E). The transmission electron microscopy (TEM) images revealed that the CS@KET/P780 nanoparticles possess a totally spherical morphology, indicating an effective coating of CS onto the surface of the KET/P780 nanoparticles (Figure 1F,G). Hyaluronidase (HY) is a glycoprotein that enzymatically cleaves the β‐acetylhexosamine‐glucoside bonds presented in hyaluronic acid, chondroitin, and CS.^[^ ^17^ ^]^ To investigate this phenomenon, a specific quantity of HY was incorporated into CS@KET/P780 NPs under acidic conditions and subsequently examined using TEM. The resulting TEM image in Figure S3E, Supporting Information, revealed that the dissociated CS@KET/P780 NPs exhibited an irregular morphology with scattered composition. Simultaneously, a positive Zeta potential is seen following the dissociation of CS@KET/P780 NPs (Figure S3F, Supporting Information). In addition, the FTIR spectra of P780, KET/P780 NPs, and CS@KET/P780 NPs also demonstrated the successful synthesis of nanomaterials (Figure 1H). After 7 d, the size fluctuation and storage instability of the CS@KET/P780 NPs were negligible, suggesting the excellent stability of CS@KET/P780 NPs (Figure 1I,J). Furthermore, dynamic light scattering (DLS) analysis was used to assess the stability of CS@KET/P780 nanoparticles suspended in DMEM medium containing 10% fetal bovine serum (FBS) and PBS over a 7 d period. As shown in Figure 1K,L, CS@KET/P780 NPs showed little change in size, with only a slight increase in both media. These results indicated that CS@KET/P780 NPs not only have better serum stability but also have better long‐term stability in PBS.

Furthermore, KET content in CS@KET/P780 NPs was determined by high‐performance liquid chromatography (HPLC), and P780 content in CS@KET/P780 NPs was investigated by UV–vis spectrophotometry (Figure S3G,H, Supporting Information). The KET and P780 loading efficiency of CS@KET/P780 NPs were calculated as 26.2% and 28.5%, respectively. In addition, the KET and P780 encapsulation efficiency of CS@KET/P780 NPs were calculated at 58.5% and 63%, correspondingly. The release behavior of P780 in CS@KET/P780 nanoparticles was measured at 37 °C after 48 h of incubation. Standard curves of P780 at pH = 7.4 and pH = 5.0 are shown in Figure S3I,J (Supporting Information). It is important to note that superior release results are observed at a pH of 5.0, indicating that CS@KET/P780 NPs have the ability to deliver pH‐responsive drugs (Figure 1M).

Subsequently, the capacity of photothermal has been assessed using laser irradiation. (1.0 W cm^−2^, 808 nm for IR780, 660 nm for P780, and CS@KET/P780 NPs). As depicted in Figure 1N,O and Figure S3K,L, Supporting Information, CS@KET/P780 NPs exhibited pronounced heating properties in both a combination of time‐ and dose‐dependent manners similar to free IR780 and P780. In addition, the photothermal conversion efficiency of CS@KET/P780 NPs was 26.8% (Figure 1P,Q). Following this, a probe known as 1,3‐diphenylisobenzofuran (DPBF) was employed to assess the photodynamic effectiveness of CS@KET/P780 NPs. The observation of identical leftover DPBF absorbance levels for KET/P780 NPs, free IR780, and P780 following 2.5 min of NIR laser irradiation suggests that KET/P780 NPs exhibit effective production of ROS (Figure 1R and Figure S3M–Q, Supporting Information). The marginally lower generation and release of ROS in the group of CS@KET/P780 NPs (Figure S3Q, Supporting Information) could be explained by the possibility that alteration of CS may have a negative impact on the interaction between P780 and O_2_. In conclusion, it could be inferred that CS@KET/P780 NPs possess the potential to serve as a very effective nanoplatform for phototherapy.

### 2.2 Cellular Uptake and Cytotoxicity Evaluation of CS@KET/P780 NPs

**Figure 2:**
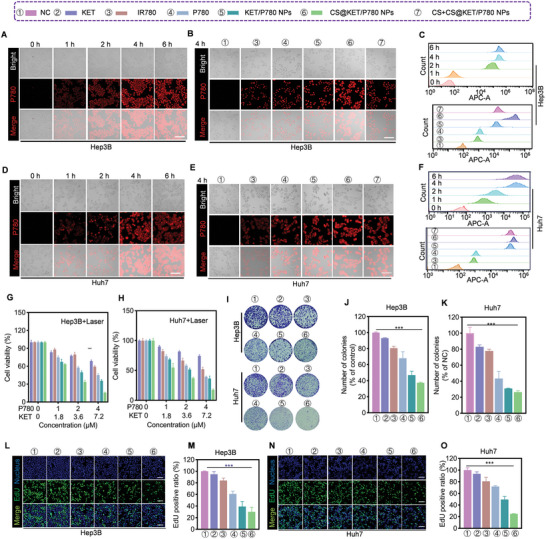
The cellular uptake and cytotoxicity of CS@KET/P780 NPs in vitro. (The following experimental conditions are: 808 nm for IR780, 660 nm for P780, KET/P780 NPs and CS@KET/P780 NPs; *P* = 1.0 W cm^−2^, irradiation time = 30 s; *C*_KET_ = 4.5 × 10^−6^ m, C_P780_ = 2.5 × 10^−6^ m). A,D) Fluorescence microscopy images of the cellular absorption of CS@KET/P780 NPs at various periods in Hep3B cells and Huh7 cells. Scale bar: 100 µm. B,E) Fluorescence microscopy images of the cellular uptake of IR780, P780, KET/P780 NPs, CS@KET/P780 NPs, and CS+ CS@KET/P780 NPs (cells were treated with CS for half an hour beforehand) in Hep3B and Huh7 cells following 4 h incubation. Scale bar: 100 µm. C,F) Results of cellular absorption in Hep3B and Huh7 cells using flow cytometry. G,H) Viability of Hep3B and Huh7 cells treated with KET, IR780, P780, KET/P780 NPs, and CS@KET/P780 NPs after NIR laser irradiation. I–K) Representative images of Hep3B and Huh7 cells after various treatments for colony formation and quantitative analysis. L,N) Fluorescence imaging and M,O) quantification analysis of EdU labeling assay in Hep3B and Huh7 cells under various treatments. Scale bar: 50 µm. (**P < 0.01; ***P < 0.001, one‐way ANOVA).

The process of efficient internalization plays a crucial role in the accumulation of drugs, which is essential for effectively suppressing tumors.^[^ ^18^ ^]^ Therefore, we chose to utilize two liver cancer cell lines of human origin (Hep3B and Huh7) and one liver cancer cell line of mouse origin (Hepa1‐6) to assess the cellular uptake efficiency of CS@KET/P780 NPs. Results from fluorescent imaging and cytometry showed that CS@KET/P780 NPs could efficiently enter tumor cells, accumulating to their greatest levels intracellularly for both Hep3B (**Figure** **2**A,C) and Huh7 cells (Figure 2D,F) after a 4 h incubation. In contrast, it was observed that the Hepa1‐6 cells achieved their highest level of uptake after a period of 6 h of incubation (Figure S4A,C, Supporting Information). Consequently, subsequent cellular investigations involving Hep3B/Huh7 and Hepa1‐6 were conducted using laser irradiation following incubation periods of 4 and 6 h, respectively. Additionally, CS@KET/P780 NPs exhibited stronger fluorescence intensity in Hep3B (Figure 2B,C), Huh7 (Figure 2E,F), and Hepa1‐6 cells (Figure S4B,C, Supporting Information) than free IR780, P780, KET/P780 NPs, and CS+CS@KET/P780 NPs, demonstrating that CS modification enhanced CS@KET/P780 NPs' cellular absorption effectiveness.

Following this, the cytotoxicity of CS@KET/P780 NPs on liver cancer cells was evaluated using the 3‐(4,5‐dimethylthiazole‐2‐yl)−2,5‐diphenyl tetrazole bromide (MTT) assay. Although the toxicity of these groups in the absence of NIR laser irradiation was not readily apparent (Figure S4E–G, Supporting Information), NIR irradiation revealed a considerably concentration‐dependent response (Figure 2G,H and Figure S4D, Supporting Information). The combination of PDT, PTT, and chemotherapy appears to have a synergistic effect, which can be related to the inclusion of photosensitizer and chemotherapeutic compounds within the nanoparticles. Furthermore, to evaluate the cytotoxicity of CS@KET/P780 NPs in normal cells, we selected immortalized human liver cells (LO2 cells) and human renal epithelial cells (293 T cells) as normal cell models to investigate the cytotoxicity experiments of CS@KET/P780 NPs. As shown in Figure S4H, Supporting Information, CS@KET/P780 NPs had low cytotoxic effects on both normal cells, indicating that CS@KET/P780 NPs could selectively destroy tumor cells while having few negative effects on normal cells. Moreover, to undertake a more comprehensive assessment of the antiproliferative effects of CS@KET/P780 NPs, colony formation and EdU tests were performed. The findings from the colony formation assay (Figure 2I–K, Figure S4I,J, Supporting Information) and EdU assay (Figure 2L–O, Figure S4K,L, Supporting Information) align with the results obtained from the MTT experiment. All things considered, our research indicates that the CS@KET/P780 NPs are remarkably effective at stopping the growth of tumors through a synergistic combination of chemotherapy and PDT/PTT, as demonstrated in an in vitro setting.

### 2.3 CS@KET/P780 NPs Evoke Apoptosis through ROS Accumulation

**Figure 3:**
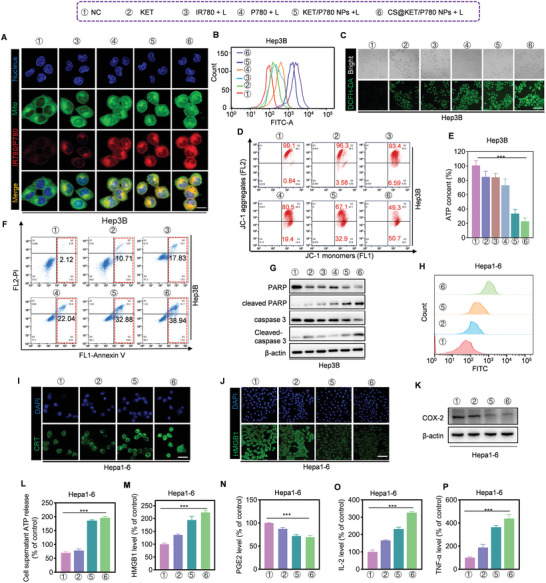
CS@KET/P780 NPs evoke apoptosis through ROS accumulation and trigger ICD in liver cancer cells (The following experimental conditions are: 808 nm for IR780, 660 nm for P780, KET/P780 NPs, and CS@KET/P780 NPs; *P* = 1.0 W cm^−2^, irradiation time = 30 s; *C*_KET_ = 4.5 × 10^−6^ m, *C*_P780_ = 2.5 × 10^−6^ m). A) LSCM images to display subcellular localization of IR780 or P780 with mitochondria. Scale bar:10 µm. B) Flow cytometry analysis and C) fluorescence imaging for intracellular ROS level of Hep3B cells using DCFH‐DA as a probe. Scale bar:100 µm. D) Flow cytometry analysis for mitochondrial membrane potential of Hep3B cells following different treatments. E) ATP content in Hep3B cells following different treatments. F) Flow cytometry findings of apoptosis in Hep3B cells after various treatments. G) Western blot analysis of apoptotic markers for Hep3B cells following different treatments. H,I) Flow cytometry evaluation and immunofluorescence staining of the CRT on the surface of Hepa1‐6 cells following various treatments. Scale bar:10 µm. J) Hepa1‐6 cells were labeled with HMGB1 immunofluorescence following various treatments. Scale bar: 60 µm. K) Western blot analysis of the COX‐2 in Hepa1‐6 cells following various treatments. L) ATP content in the supernatant of Hepa1‐6 cells after various treatments. M) HMGB1, N) PGE2, O) IL‐2, and P) TNF‐α in the supernatant of Hepa1‐6 cells after various treatments. (***P < 0.001, one‐way ANOVA).

Mitochondria are responsible not only for energy production but also for maintaining redox metastasis.^[^ ^19^ ^]^ Therefore, it is promising that targeting mitochondria and mitochondrial‐derived ROS levels might be an effective strategy for suppressing tumor growth. Firstly, we investigated the mitochondrial‐targeting property of CS@KET/P780 NPs using the Mito‐Tracker Green FM (MTG) probe. The red and green fluorescence represent IR780/P780 and the mitochondria, respectively. Upon co‐localization of the nanoplatform with mitochondria, the fluorescence emitted by IR780/P780 and the fluorescence emitted by mitochondria were shown to combine, resulting in the manifestation of yellow fluorescence. As shown in **Figure** **3**A and Figure S5A–E, Supporting Information, P780 exhibited better colocalization with mitochondria than IR780, and the CS@KET/P780 NPs group showed the most accumulation in the mitochondria, confirming the good mitochondria‐targeting ability of CS@KET/P780 NPs.

Then, to assess the intracellular generation of ROS induced by CS@KET/P780 nanoparticles, a fluorescent probe known as 2,7‐dichlorodihydrofluorescein diacetate (DCFH‐DA) was utilized for the quantification of ROS level using fluorescence imaging and flow cytometry (Figure 3B,C and Figure S5F–I, Supporting Information). Following laser irradiation, the CS@KET/P780 NPs exhibited the highest level of green fluorescence in contrast to free KET, IR780, P780, KET/P780 NPs, and PBS, indicating a powerful photodynamic effect for cancer treatment. To conduct a more comprehensive examination of the role of ROS, the utilization of N‐acetyl cysteine (NAC), a scavenger of ROS, was implemented to mitigate the elevated levels of ROS (Figure S5J–L, Supporting Information). Moreover, the cytotoxicity phenotype was also ameliorated by NAC in the MTT experiment (Figure S5M–O, Supporting Information), which further proved the ROS‐induced therapeutic effect of CS@KET/P780 NPs in liver cancer.

Previous studies have demonstrated that increased heat and ROS could result in mitochondrial damage.^[^ ^20^ ^]^ The utilization of the JC‐1 dye enabled the assessment of alterations in mitochondrial membrane potential, hence providing an effective evaluation of mitochondrial activity. As anticipated, the utilization of CS@KET/P780 NPs resulted in a significant transformation of JC‐1 aggregates into monomers, as evidenced by the findings from flow cytometry and fluorescence imaging (Figure 3D and Figure S6A–E, Supporting Information). These observations provide strong evidence for a reduction in mitochondrial membrane potential and the occurrence of mitochondrial dysfunction. We next investigated the intracellular ATP level in Hep3B, Huh7, and Hepa1‐6 cells after different treatments. Consequently, CS@KET/P780 NPs exhibit a clear reduction in intracellular ATP levels compared to other groups, hence confirming the potent inhibitory impact of CS@KET/P780 NPs on ATP synthesis. (Figure 3E and Figure S6F,G, Supporting Information).

There is evidence that high intracellular ROS buildup can cause cancer cells to undergo apoptosis.^[^ ^21^ ^]^ Unsurprisingly, CS@KET/ P780 NPs greatly promoted apoptosis ratios in Hep3B cells (Figure 3F), Huh7 cells (Figure S7A, Supporting Information), and Hepa1‐6 cells (Figure S7C, Supporting Information) using Annexin V/PI flow cytometry staining. Additionally, increased expression of apoptotic markers (e.g., cleaved‐ PARP, cleaved‐caspase 3) was observed in CS@KET/P780 NPs‐treated cells (Figure 3G and Figure S7B,D, Supporting Information), which is consistent with the flow cytometry results. Furthermore, the augmented rate of apoptosis following treatment with CS@KET/P780 NPs was ameliorated through the administration of NAC, suggesting the involvement of ROS in the induction of apoptosis by CS@KET/P780 NPs (Figure S7E,F, Supporting Information). It is worth noting that the viability of cells can be restored following treatment with zVAD, as depicted in Figure S7G of the Supporting Information. This finding provides another evidence to support the notion that CS@KET/P780 nanoparticles exhibit anti‐tumor properties in liver cancer through the induction of ROS buildup and subsequent apoptosis.

Notably, our previous study investigated mitochondrial autophagy induced by ketoconazole in HCC cells.^[^ ^7^ ^]^ Therefore, we explored whether CS@KET/P780 NPs induce autophagy in HCC cells. The study showed a decrease in the accumulation of the autophagy‐specific substrate SQSTM1 (p62) and an elevation in microtubule‐associated protein light chain 3 (LC3)‐II levels in HCC cells treated with CS@KET/P780 NPs. (Figure S7H–J, Supporting Information). These findings indicated that mitochondria‐targeting CS@KET/P780 NPs evoked hyperthermia and ROS generation when exposed to laser irradiation, leading to apoptosis caused by mitochondrial dysfunction.

### 2.4 CS@KET/P780 NPs Trigger Immune Response of HCC In Vitro

There is a growing body of evidence indicating that several therapeutic approaches, including radiotherapy, chemotherapy, and photodynamic therapy (PDT), have the potential to induce apoptosis, thereby efficiently initiating ICD.^[^ ^22^ ^]^ Recently, it has been revealed that cycle oxidase‐2 and prostaglandin E2 (PGE2) expression in tumor cells can promote cancer immune escape.^[^ ^23^ ^]^ These factors collectively facilitate the infiltration of innate immune cells into the afflicted tissue for the purpose of eliminating diseased cells and initiating the subsequent immune maturation process to elicit immunity.

Given the observed apoptotic properties of CS@KET/P780 nanoparticles, it is imperative to conduct additional investigations to validate the in vitro immune response elicited by CS@KET/P780 NPs. Subsequently, the expression levels of CRT, HMGB1, and ATP secretion were quantified in HCC cells following treatment with PBS, KET, KET/P780 NPs, and CS@KET/P780 NPs. The immunofluorescence images and flow cytometry analysis (Figure 3H,I) showed that cell‐surface CRT expression increased in the CS@KET/P780 NPs‐treated cells, and after treatment with CS@KET/P780 NPs, the fluorescence of HMGB1 in the nucleus was attenuated, and HMGB1 translocated into the cytoplasm (Figure 3J). Moreover, we used western blotting to analyze the ability of CS@KET/P780 NPs to down‐regulate COX‐2 in liver cancer cells. As shown in Figure 3K, CS@KET/P780 NPs significantly reduced COX‐2 expression in cells. Afterwards, the amount of ATP in the cell supernatant was detected with an ATP detection kit after different treatments. As indicated in Figure 3L, the level of ATP release in the extracellular region increased significantly in the CS@KET/P780 NPs group. Additionally, CS@KET/P780 NPs treatment significantly enhanced the extracellular levels of HMGB1, PGE2 and pro‐inflammatory cytokines (IL‐2, TNF‐α) in Hepa1‐6 cells measured by enzyme‐linked immunosorbent assay (ELISA) (Figure 3M–P). These findings clearly demonstrated that CS@KET/P780 NPs have the capacity to both significantly limit tumor formation by lowering COX‐2 and PGE2 production and to provide a notable ICD effect in vitro when used in conjunction with immunotherapy.

### 2.5 In Vivo Biodistribution and Antiliver Cancer Performance of CS@KET/P780 NPs in BALB/c Nude Mice

A hemolytic assay was conducted to investigate the blood compatibility of CS@KET/P780 NPs prior to intravenous administration (Figure S8A, Supporting Information). The as‐obtained samples' hemolytic rates within the tested concentrations were less than 5%, indicating that CS@KET/P780 NPs are feasible for intravenous delivery.^[^ ^24^ ^]^

**Figure 4:**
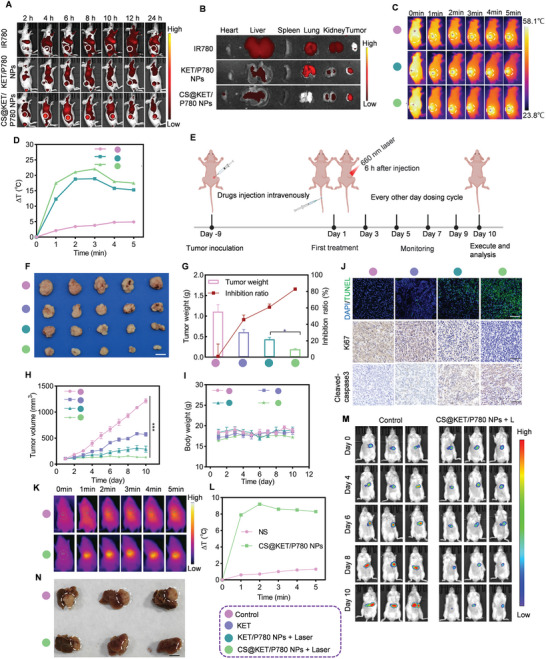
In vivo biodistribution and anti‐liver cancer performance of CS@KET/P780 NPs in BALB/c nude mice. The mice were treated with normal saline, free KET, KET/P780 NPs with laser irradiation, or CS@KET/P780 NPs with laser irradiation (The following experimental conditions are: *λ* = 660 nm, *P* = 1.0 W cm^−2^; irradiation time = 3 min; *C*_KET_ = 2 mg kg^−1^, *C*_P780_ = 2 mg kg^−1^, *V* = 100 µL). A) Real‐time in vivo imaging of IR780, KET/P780 NPs, and CS@KET/P780 NPs following intravenous injection in the Hep3B tumor‐bearing BALB/c nude mice model. B) Analysis of radiance intensity of dissected major organs and tumors at 24 h postadministration. C) Images captured when BALB/c nude mice were exposed to NIR laser radiation at various time intervals at tumor locations. The white circles represent tumor sites. (*P* = 1.0 W cm^−2^ for 0, 1, 2, 3, 4, and 5 min). D) Temperature‐change profiles at the tumor sites following intravenous administration. E) Schematic representation of the design and procedure of tumor treatment of the Hep3B tumor‐bearing BALB/c nude mice model. Created with BioRender.com. F) Representative pictures of isolated tumors. Scale bar: 0.5 cm. G) The tumor weight of different groups and the inhibition rate. H) The tumor volume of mice in each group and I) the corresponding body weight were measured at indicated time points (*n* = 5). Scale bar: 50 µm. J) TUNEL and immunohistochemical staining of cleaved‐caspase 3 and Ki67 in tumor tissues after various strategies. K) Images captured when NSG mice were exposed to NIR laser radiation at various time intervals at tumor locations. The red circles represent tumor sites. (*P* = 1.0 W cm^−2^ for 0, 1, 2, 3, 4, and 5 min). L) Temperature‐change profiles at the tumor sites following intravenous administration. M) In vivo bioluminescence images of the orthotopic Hep3B‐Luc tumors at different time intervals from Day 0 to Day 10. N) Digital photo of the isolated tumor tissues from the liver after various treatments on Day 10. The reddish‐brown tissue is the residual liver. The white tissue is the tumor. Scale bar: 0.5 cm. (***P < 0.001, one‐way ANOVA).

A near‐infrared fluorescence imaging system was utilized to track the fluorescence signal of CS@KET/P780 NPs at different time intervals in the subcutaneous tumor‐harboring BALB/c mice model in order to evaluate the biodistribution and tumor‐specific accumulation of these nanoparticles (**Figure** **4**A). Fluorescence imaging indicated CS@KET/P780 NPs exhibited stronger fluorescence intensity in the tumor region and better tumor accumulation ability than free IR780 and KET/P780 NPs, which may benefit from the presence of CS. Moreover, at 6 h postinjection, the CS@KET/P780 NPs groups showed the strongest fluorescence signal in the tumor site. Consequently, subsequent studies were conducted by employing near‐infrared laser irradiation for 6 h following intravenous delivery. Furthermore, significant organs and tumor tissues were obtained for ex vivo imaging 24 h after injection. As shown in Figure 4B, the fluorescence accumulation time of CS@KET/P780 NPs within tumor tissues exhibited a prolonged duration, indicating enhanced drug aggregation for efficient chemo‐phototherapy. The thermal pictures obtained from the NIR spectrum and the heating curves were analyzed at 6 h postinjection. The normal saline group saw little temperature change at the tumor location following NIR laser irradiation, as illustrated in Figure 4C,D. By contrast, the surface temperature at the tumor site in the group treated with CS@KET/P780 NPs exhibited an increase of approximately 20 °C (3 min postirradiation), surpassing the temperature observed in the group treated with KET/P780 NPs. These results indicated that the use of CS exhibited remarkable active targeting capabilities and increased the efficacy of photothermal therapy.

To further evaluate the therapeutic effect of CS@KET/P780 NPs in vivo, we successfully established Hep3B cells model of a subcutaneous tumor (Figure 4E). Following a 9 d period of inoculation with Hep3B cells, mice were divided into four groups in a random manner. Each group was given different reagents every 2 d for 10 d through the tail vein: I) normal saline (NS), II) KET, III) KET/P780 NPs, and IV) CS@KET/P780 NPs. 6 h postinjection, the tumor was irradiated with 660 nm laser (1.0 W cm^−2^) for 3 min. It was found that CS@KET/P780 NPs exhibited much better therapeutic effects than other treatment groups (Figure 4F–H). Immunohistochemistry (IHC) and TUNEL staining of tumor tissues further verified the remarkable anticancer impact of CS@KET/P780 NPs by inducing apoptosis and inhibiting cell proliferation (Figure 4J). In addition, no noticeable weight changes were observed in the tumor‐bearing mice with different treatment groups, indicating the good biosafety of the nanoparticles (Figure 4I). Moreover, serum biochemical indicators (ALT, AST, CRE, and UREA) showed negligible fluctuation under CS@KET/P780 NPs treatment (Figure S8B–E, Supporting Information). H&E staining of the major organs (Figure S8F, Supporting Information) exhibited negligible systemic toxicity after CS@KET/P780 NPs administration. However, notable harm to tumor tissue was found in the CS@KET/P780 NPs group when subjected to NIR laser irradiation. These results demonstrated that CS@KET/P780 NPs had antitumor potential, good biosafety, and possessed promising potential for HCC therapy.

### 2.6 In Vivo Antiliver Cancer Performance of CS@KET/P780 NPs on Orthotopic Hepatoma Model

Encouraged by the excellent tumor inhibition activity of the CS@KET/P780 NPs on the subcutaneous xenograft tumor model, an orthotopic hepatoma model was further fabricated to investigate the antitumor performance. In this work, Hep3B cells transfected with the Luciferase gene were utilized to create in‐situ liver cancer models in extremely immunodeficient mice (NSG mice), allowing bioluminescence imaging to track the growth of the tumors. NSG mice bearing orthotopic Hep3B‐Luc cells were randomly separated into two groups for injection of two reagents comprising i) normal saline and ii) CS@KET/P780 NPs on days 1, 3, 5, 7, and 9. The thermal pictures obtained from the NIR spectrum and the heating curves were analyzed at 6 h postinjection. As shown in Figure 4K,L, the normal saline group exhibited minimal temperature fluctuations at the tumor site following NIR laser exposure. In contrast, the group that received CS@KET/P780 NPs treatment showed a rise in surface temperature at the tumor location of around 10 °C within 3 min postirradiation, suggesting that CS@KET/P780 NPs exhibited excellent photothermal therapy efficacy in the orthotopic hepatoma model. Furthermore, tumor progression was detected on days 0, 4, 6, 8, and 10 using IVIS spectrum imaging equipment, as depicted in Figure 4M. When subjected to laser radiation, the CS@KET/P780 NPs group exhibited significant tumor inhibition ability, as evidenced by the minimal bioluminescence signal observed in the in vivo bioluminescence imaging results. This observation was further supported by the consistent findings in the photograph of *ex vivo* tumor tissues (Figure 4N). These findings showed that CS@KET/P780 NPs have excellent potential for treating HCC and exhibited good anticancer potential for primary liver cancer.

### 2.7 In Vivo Biodistribution and Antiliver Cancer Performance of CS@KET/P780 NPs in C57BL/6 Mice

**Figure 5:**
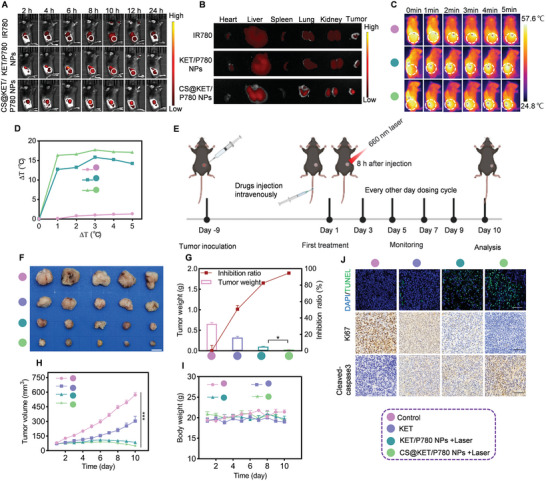
In vivo biodistribution and anti‐liver cancer performance of CS@KET/P780 NPs in C57BL/6 mice. The mice were treated with normal saline, free KET, KET/P780 NPs with laser irradiation, or CS@KET/P780 NPs with laser irradiation (The following experimental conditions are: *λ* = 660 nm, *P* = 1.0 W cm^−2^; irradiation time = 3 min; *C*_KET_ = 2 mg kg^−1^, *C*_P780_ = 2 mg kg^−1^, *V* = 100 µL). A) The performance of CS@KET/P780 NPs against liver cancer in vivo in C57BL/6 mice. B) The fluorescence intensity of dissected primary organs and tumors at 24 h after treatment. C) In vivo thermal infrared pictures of C57BL/6 mice model with laser irradiation at tumor sites at various time intervals. The white circles represent tumor sites (0, 1, 2, 3, 4, and 5 min). D) Temperature‐change profiles at the tumor sites following intravenous administration. E) Schematic representation of the design and procedure of tumor treatment of the Hepa1‐6 tumor‐bearing C57BL/6 mice model. Created with BioRender.com. F) Representative pictures of isolated tumors. Scale bar: 0.5 cm. G) The tumor weight of the different groups and the inhibition rate. H) The tumor volume of mice in each experimental group and I) the corresponding body weight were measured at indicated time points (*n* = 5). J) TUNEL and immunohistochemical staining of Ki67 and cleaved‐caspase 3. Scale bar: 50 µm. (***P < 0.001, one‐way ANOVA).

Based on the good antitumor effect of CS@KET/P780 NPs on BALB/c nude mice, we next selected C57BL/6 mice with healthy immune systems to verify the anti‐tumor effect of CS@KET/P780 NPs. In vivo imaging showed that CS@KET/P780 NPs had higher tumor accumulation ability than IR780 and KET/P780 NPs, and the fluorescence intensity reached the strongest signal at 8 h after injection (**Figure** **5**A). Essential organs and tumor tissue were then excised for the purpose of investigating the efficacy of accumulation. CS@KET/P780 NPs demonstrated a stronger fluorescence signal at the tumor site than IR780 and KET/P780 NPs, as seen in Figure 5B. The NIR thermal pictures and temperature curves were analyzed at 8 h postinjection (Figure 5C,D). These findings suggest that the CS@KET/P780 NPs possess excellent targeting ability and demonstrate remarkable photothermal performance in the C57BL/6 mouse model. Follow‐up experiments were conducted using NIR laser irradiation for 3 min and 8 h after tail vein injection. In order to conduct a comprehensive assessment of the therapeutic efficacy of CS@KET/P780 NPs in an in vivo setting, a subcutaneous tumor model was successfully established with Hepa1‐6 cells (Figure 5E). As shown in Figure 5F–J, the IHC results, the observation of tumor tissue visually, tumor volume, body weight change, and tumor inhibition rate of mice were consistent with the tumor inhibition results in BALB/c nude mice.

Furthermore, to evaluate the antitumor effects of CS@KET/P780 NPs with or without laser treatments, the tumor‐bearing mice were randomly assigned to three groups: normal saline (NS) group, CS@KET/P780 NPs group, and CS@KET/P780 NPs + laser group. Each mouse received an equivalent volume of either NS or CS@KET/P780 NPs via tail vein administration every 2 d for 10 d. Subsequently, 8 h postinjection, the mice in the CS@KET/P780 NPs + laser group underwent irradiation with a 660 nm laser (1.0 W cm^−2^) for 3 min at the tumor site. As shown in Figure S9A (Supporting Information), a notable decrease in tumor volume was observed in the group treated with CS@KET/P780 NPs + laser, which exhibited a significantly smaller tumor volume compared to the group treated with CS@KET/P780 NPs alone. Specifically, the tumor inhibition ratios for the groups treated with NS, CS@KET/P780 NPs, and CS@KET/P780 NPs + laser were approximately 1.11%, 37.54%, and 95%, respectively (Figure S9B, Supporting Information). Moreover, the images of the excised tumors from the different groups were consistent with the observed tumor inhibition effects (Figure S9C, Supporting Information). These results demonstrated the CS@KET/P780 NPs + laser group was very useful for fighting tumors.

### 2.8 CS@KET/P780 NPs Induces Antitumor Immunological Responses In Vivo

**Figure 6:**
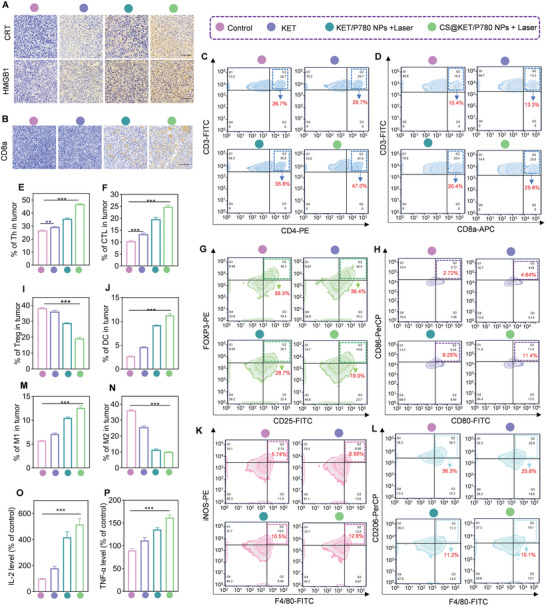
The CS@KET/P780 NPs‐mediated immune activation in vivo. The mice were treated with normal saline, free KET, KET/P780 NPs with laser irradiation, or CS@KET/P780 NPs with laser irradiation (*λ* = 660 nm, *P* = 1.0 W cm^−2^; irradiation time = 3 min; *C*_KET_ = 2 mg kg^−1^, *C*_P780_ = 2 mg kg^−1^, *V* = 100 µL). A) Immunohistochemical tests of CRT and HMGB1 in tumor tissues after various therapies. B) Immunohistochemical analysis of CD8a in tumor tissues after various therapies. C–P) Flow cytometry analysis and quantitative analysis of the percentage of major immune cells in tumors of mice, including C,E) CD4^+^ T cells, D,F) CD8^+^ T cells, G,I) Tregs, H,J) DCs, K,M) M1 macrophage and L,N) M2 macrophage. O,P) IL‐2 and TNF‐α content in mice serum following different treatments detected by ELISA. All data are presented as the means ± SD from at least 3 independent experiments. (**P<0.01, ***P<0.001, one‐way ANOVA).

To confirm that apoptosis and phototherapy trigger anti‐tumor immunity via ICD in vivo, CRT exposure and HMGB1 release were evaluated by IHC. As shown in **Figure** **6**A, more significant increase of CRT and HMGB1 was found in the CS@KET/P780 NPs group than either the NS and KET groups, which was consistent with the results of in vitro analysis. What is more, infiltration and activation of antigen‐specific CD8^+^ and CD4^+^ T cells within tumors were detected in C57BL/6 mice models. As expected, CS@KET/P780 NPs treatment promoted significant infiltration of CD4^+^ (Figure 6C,E) and CD8^+^ (Figure 6B,D,F) T cells within tumors. Tumor growth is aided by Treg cells, a highly immunosuppressive subgroup of CD4^+^ T cells.^[^ ^25^ ^]^ We therefore also measured the proportion of Treg cells within tumors. The results showed that CS@KET/P780 NPs treatment significantly decreased the percentage of Treg cells, suggesting CS@KET/P780 NPs could overcome the immunosuppressive microenvironment (Figure 6G,I). DCs are critical in antigen presentation to reduce adaptive anti‐tumor immunity.^[^ ^26^ ^]^ The proportion of mature DCs was further quantified (Figure 6H,J). The percentage of mature DCs (CD11c^+^CD80^+^CD86^+^) in KET/P780 NPs and CS@KET/P780 NPs groups were 9.25% and 11.4%, respectively, both of which are higher than the control group (2.72%). These findings demonstrated that CS@KET/P780 NPs produced a systemic immune response.

It has been demonstrated that promoting effective macrophage polarization from the M2 to M1 phenotype holds significant potential for enhancing tumor immunotherapy.^[^ ^27^ ^]^ As shown in Figure 6K–N, treatment with CS@KET/P780 NPs could achieve macrophage polarizations from the M2 to M1 phenotype. Serum analysis revealed that the levels of IL‐2 and TNF‐αwere increased by the CS@KET/P780 NPs treatment (Figure 6O,P). Collectively, CS@KET/P780 NPs upregulated immunosurveillance cells and downregulated immunosuppressive cells, thereby effectively activating systemic immune response based on ICD.

## 3 Conclusion

In summary, we successfully designed a pH‐sensitive and mitochondria‐targeting nanoplatform to deliver KET and IR780 to liver cancer cells. The nanoplatform enhanced the bioavailability of KET and amplified photochemotherapy, thereby triggering ICD and activating the systemic immune response. In vivo results demonstrated that CS@KET/P780NPs treatment could effectively inhibit the growth of HCC cells. Further studies showed that CS@KET/P780NPs effectively activated the immune system in vivo and reshaped the immunosuppressive microenvironment within tumors. The current design exhibits three advantages. CS@KET/P780NPs solved the poor solubility and bioavailability of KET. Besides, CS@KET/P780NPs may accomplish specific mitochondrial targeting in tumor cells following modification with TPP. After lysis in an acidic tumor environment, KET/P780NPs rapidly accumulated in the mitochondria, releasing deadly ROS that effectively caused mitochondrial malfunction and intrinsic tumor cell apoptosis. Finally, our photochemotherapy platform may considerably boost the immune response by enhancing the immunosuppressive environment thanks to the in situ amplification of both PTT and PDT by mitochondrial targeting, displaying exceptional anti‐tumor immunotherapy impact. Moreover, CS@KET/P780NPs showed good biosafety, with no obvious harm to vital organs, suggesting their potential application in the treatment of HCC. Therefore, this work indicates that CS@KET/P780NPs is a promising antitumor agent that can target mitochondria and enhance photochemotherapy, providing a promising clinical treatment strategy for the efficient treatment of tumors.

## 4 Experimental Section

### Materials

Ketoconazole (Catalog no. K1003) was purchased from MedChemExpress, IR780 iodide (Catalog no. 207399‐07‐3), 3‐bromopropylamine hydrobromide, hyaluronidase (Catalog no.37326‐33‐3), and triphenylphosphine were obtained from J&K Scientific. 1,3‐Diphenylisobenzofuran (DPBF), dimethyl sulfoxide (DMSO), chondroitin sulfate (Catalog no. 24967‐93‐9), and crystal violet (Catalog no. C0775) were purchased from Aladdin Industrial Corporation. The One Step TUNEL Apoptosis Assay Kit was purchased from Beyotime Biotechnology. Mito‐Tracker Green and Hoechst 33342 were bought from Life Technologies. Penicillin and streptomycin were acquired from Millipore Sigma. RiboBio Co. supplied the EdU labeling test kit (C10310‐1). Mouse IL‐2 ELISA Kit (EK202/2‐01), and mouse TNF‐alpha ELISA Kit (GEM0004) was received from Elabscience. The types of antibodies used are as follows: PARP, caspase 3, and p62 were acquired from Cell Signaling Technology; LC3 was received from Novus. CRT Rabbit Monoclonal Antibody (WL04297), Ki67 (bsm‐33 070 M), and HMGB1 Rb pAb (WL03023) were obtained from WanLeibio.

### Synthesis and Verification of TPP‐NH_2_ and P780 (TPP‐NH_2_‐IR780)

In step 1: On the basis of the reported method of synthesizing TPP‐NH_2_,^[^ ^28^ ^]^ the target product was finally got by optimizing the experimental steps. Briefly, triphenylphosphine (0.8741 g, 3.3 mmol) and 3‐bromomylamine hydrobromide (0.7297 g, 3.3 mmol) were added to 15 mL of anhydrous acetonitrile in a round‐bottomed flask. The resultant mixture was then refluxed in an oil bath at 80–85 °C for 16 h. After stirring, the mixture was cooled to ambient temperature, and the anhydrous acetonitrile solvent was then spun out to produce crude TPP‐NH_2_. The white product was obtained by recrystallization of the crude product with a mixture of ethyl ether (12.5 mL), isopropanol (25 mL), n‐hexane (7.5 mL), and acetonitrile (2.5 mL) at 4 °C.

In step 2: In a 50 mL round‐bottom flask, IR780 iodide (0.1667 g, 0.25 mmol) was completely dissolved in anhydrous N, N‐dimethyl formamide (2.5 mL), and dichloromethane (2.5 mL). Subsequently, under magnetic stirring, 2.5 mL of 0.5 mmol TPP‐NH_2_ (blended with anhydrous DMF) and 137 µL of triethylamine were added dropwise. In an oil bath heated to 85 °C and shielded from light for 6 h, the reaction was stirred. Following the removal of DMF, column chromatography was used to purify the crude product using methylene chloride and methanol as eluents. The structures of the products in step 1 and step 2 were analyzed by UV–vis spectrophotometry,^1^ H NMR, ^13^C NMR and HR‐MS.

### Synthesis and Characterization of CS@KET/P780 NPs

KET (1 mg) and P780 (1 mg) were separately dissolved in methanol (MeOH, 100 µL), and the two were mixed thoroughly by ultrasound. The prepared mixture was slowly poured into 2 mL of deionized water containing CS while being stirred at 1000 rpm. The methanol was eliminated by rotary evaporation following 30 min of agitation, and the CS@KET/P780 NPs product was produced. In addition, other CS@KET/P780 NPs with various mass ratios were obtained similarly as mentioned above. Briefly, different weights of CS or P780 were dissolve in ddH_2_O or MeOH (CS/P780, 0:1, 1:1, 2:1, 3:1, 4:1, w/w) or (KET/P780, 1:1, 1:2, 1:3, 1:4, 1:5, w/w). The right amount of CS and P780 was chosen by taking into account the size distribution and zeta potential. The Zeta potential, diameter distribution, and morphology of CS@KET/P780 NPs were observed by nanoparticle tracking analyzer (NTA, PTM ZetaView) and transmission electron microscopy (Hitachi HT7800, TEM, Sichuan University). UV–vis absorption spectra of CS@KET/P780 NPs, P780, IR780, and KET were all tested using a UV–vis spectrophotometer (UV‐3600, Shimadzu, Japan). The content of P780 incorporated in CS@KET/P780 NPs was determined by UV−vis standard curves.

### The Drug Loading Contents and Encapsulation Efficiency of CS@KET/P780 NPs

The drug loading contents and encapsulation efficiency of P780 and KET in CS@KET/P780 NPs were calculated based on the following formulas after determining the concentrations of P780 and KET in CS@KET/P780 NPs via UV–vis (for P780) or HPLC (for KET) standard curves. (Analytical & Testing Center of Sichuan University).

(1) $$\mathrm{The}\;\mathrm{drug}\;\mathrm{loading}\;\mathrm{of}\;\mathrm{KET}\;\left(\%\right)=\frac{C_{\mathrm{KET}}\times V}{C_{\mathrm{KET}}\times V+C_{P780}\times V+M_{\mathrm{CS}}}\times 100\%$$

(2) $$\mathrm{The}\;\mathrm{drug}\;\mathrm{loading}\;\mathrm{of}\;P780\;\left(\%\right)=\frac{C_{P780}\times V}{C_{\mathrm{KET}}\times V+C_{P780}\times V+M_{\mathrm{CS}}}\times 100\%$$

where *C*_KET_ and *C*_P780_ referred to the concentrations of KET and P780 in CS@KET/P780 NPs, *V* referred to the volume of CS@KET/P780 NP solution, and *M*_CS_ referred to the weight of CS.

(3) $$\mathrm{Encapsulation}\;\mathrm{efficiency}\;\;\left(\%\right)=\frac{W_{a}}{W_{A}}\times 100\%$$

In this formula, *W*_a_ was the encapsulated KET and P780 amount in CS@KET/P780 NPs, and *W*_A_ was the total added KET and P780 amount.

### In Vitro Release of P780 from CS@KET/P780 NPs

The release of P780 from CS@KET/P780 NPs was measured using the dialysis technique at 37 °C and varied pH values. Briefly, KET or CS@KET/P780 NP solution (2.5 mg mL^−1^ for P780) was transferred into a dialysis bag (3500 Da molecular weight cutoff). The bag was then continuously shaken (300 rpm, 37 °C) while submerged in PBS of varied pH (7.4 or 5.0, 40 mL) containing DMF (0.10%, w/w). Then the sample (1 mL) was collected at various time intervals and detected via a UV–vis spectrometer. Meanwhile, the remaining mixture received the same volume (1 mL) of new medium.

### Photothermal Capability In Vitro

The different lasers were selected according to the maximum UV absorbance peaks of IR780, P780, and CS@KET/P780 NPs. Briefly, the sample solutions (1 mL) were exposed to 660 nm (for P780 and CS@KET/P780 NPs) or 808 nm (for IR780) laser (1.0 W cm^−2^, 5 min). By observing the temperature changes after laser exposure, the photothermal effect of IR780, P780, and CS@KET/P780 NPs at various concentrations was assessed. Moreover, thermal infrared pictures were taken at 1 min intervals using an infrared thermal imager (FOTRIC 311CE). Additionally, to assess the photothermal efficiency of CS@KET/P780 NPs, a solution containing 1 mL of CS@KET/P780 NPs was subjected to irradiation using a 660 nm laser at a power density of 1.0 W cm^−2^. The temperature was measured using a digital thermometer at specified intervals. The photothermal conversion efficiency (*η*) was calculated using the formula provided below:

(4) $$\eta \left(\%\right)=\frac{h_{S}\Delta T\max-Q_{\mathrm{DiS}}}{I\left(1-10^{-A}\lambda \right)}$$

(5) $$hs=\frac{mc}{\tau_{s}}$$

(6) $$\tau =-\frac{t}{\ln\theta }$$

(7) $$\theta =\frac{T-T\mathrm{surr}}{\Delta T\max}$$

In this context, “*h*” represented the heat transfer coefficient, “*S*” denotes the surface area of the receptacle, “Δ*T*_max_” signifies the temperature difference between the maximum system temperature and the environmental temperature, “*Q*_Dis_” represented the heat dissipated from the laser through the solvent, “*I*” denotes the laser power, and “*A*_λ_” refers to the absorbance at a specific wavelength.

### Singlet Oxygen (^1^O_2_) Quenching Capacities In Vitro

Using 1,3‐diphenylisobenzofuran (DPBF) as a probe, the singlet oxygen (^1^O_2_) quenching capacity could be detected since its absorbance value at about 420 nm would drop.^[^ ^29^ ^]^ In brief, 15 µL DPBF solution (15 µg mL^−1^, dissolved in DMF) was separately added to 1 mL of water, IR780, P780 or CS@KET/P780 NPs (containing equivalent concentration of IR780 and P780, 2.5 × 10^−6^ m). After that, all experimental groups received 660 nm (for P780 and CS@KET/P780 NPs) or 808 nm (for IR780) NIR irradiation (1.0 W cm^−2^) for 150 s. The remaining DPBF (%) was calculated based on the following formulas: the remaining DPBF (%) = *A*_t_/*A*_o_. Whereas *A*_t_ referred to the DPBF absorbance that was still present after each irradiation interval, Ao referred to the absorbance at the start.

### Evaluation of Hemolysis

The feasibility of CS@KET/P780 NPs for intravenous injection was preliminarily evaluated by hemolysis experiment. These procedures were based on the protocol in a previous paper.^[^ ^30^ ^]^ Blood was collected from the heart of New Zealand rabbits was collected, centrifuged, and washed several times to obtain erythrocyte precipitates, which were diluted into a 2% RBC suspension with normal saline. The experiment was divided into three groups as follows (3 replicates). Experimental group: CS@KET/P780 NPs of different concentrations were equally mixed with the prepared RBC suspension at a 1:1 ratio. Negative control group: normal saline and the prepared RBC suspension were averagely mixed at a 1:1 ratio. Positive control group: the prepared RBC suspension and ddH_2_O were medially mixed in a 1:1 ratio. The supernatant from the aforementioned samples was obtained by centrifuging them for 15 min at 1000 rpm after they had been in a water bath at 37 °C for 3 h. In order to determine the absorbance of the supernatant in various groups at 540 nm, a UV–vis spectrophotometer was employed. The following formula was used to calculate the hemolysis rate:

(8) $$\mathrm{Hemolysis}\;\mathrm{rate}\;\%=\frac{A_{S}-A_{N}}{A_{P}-A_{N}}\times 100\%$$

where *A*_S_, *A*_N_, and *A*_P_ were the absorbance of the experimental group, negative control group, and positive control group, respectively.

### Cell Culture

Human HCC cell lines including Huh7, Hep3B, and mouse HCC cell line Hepa1‐6 were incubated in DMEM (Thermo Fisher Scientific), supplemented with 10% FBS (Biological Industries), 100 U mL^−1^ streptomycin (HyClone) and 100 U mL^−1^ penicillin–streptomycin under a humidified incubator of 5% carbon dioxide at 37 °C.

### Analysis of In Vitro Cellular Uptake

In vitro cellular uptake of free IR780, P780, KET/P780 NPs, CS@KET/P780 NPs (with equivalent IR780 of 2.5 × 10^−6^ m and P780 of 2.5 × 10^−6^ m) at various time points was determined by fluorescent inverted microscope (Nikon, Tokyo, Japan) and flow cytometer (BD, FACS Celesta), respectively.

### In Vitro Cytotoxicity Studies and Proliferation Assays

The MTT test was used to determine the vitality of the cells treated with different concentrations of KET, IR780, P780, KET/P780 NPs, and CS@KET/P780 NPs. Briefly, Hep3B, Huh7, and Hepa1‐6 cells were grown for 24 h in 96‐well plates at a density of 4000 cells per well. Next, the cells were incubated with various concentrations of normal saline (NC), KET, IR780, P780, KET/P780 NPs, and CS@KET/P780 NPs for 4 and 6 h. Then the cells were exposed to laser radiation for 30 s at 660 nm (for P780, KET/P780 NPs, and CS@KET/P780 NPs) or 808 nm (for IR780) after discarding the growth media and rinsing them with normal saline and incubated for another 2 h. As previously reported, the absorbance was measured at a wavelength of 570 nm.^[^ ^31^ ^]^ Colony formation test, as previously described, was used to assess cell proliferation.^[^ ^32^ ^]^ In a nutshell, 500 cancer cells were cultivated in 24‐well plates before being exposed to the same reagent dose for 4 h. After saline washout, intervention was performed using 660 or 808 nm laser irradiation for 30 s (1.0 W cm^−2^). The colonies were fixed with 4% paraformaldehyde for 40 min after one week, and then stained with 0.2% crystal violet (Beyotime, C0121) for 2 h at room temperature. After drying and many washes with ultrapure water, pictures were taken, and Image J was used to calculate the percentage of clones that formed. To examine the rate of cell proliferation, the 5‐ethynyl‐2′‐deoxyuridine (EdU) test was applied. The assays were performed according to the instructions of the reagents (C10310‐1) in vitro subcellular localization.

### In Vitro Subcellular Localization of Mitochondria‐Targeted CS@KET/P780 NPs

Huh7, Hep3B, and Hepa1‐6 cells (1000 cells per well) were cultured in confocal small dishes for 24 h. Afterward, three different kinds of tumor cells were cultivated in media with the same concentration of IR780, P780, KET/P780 NPs or CS@KET/P780 NPs (2.5 × 10 m for P780 and IR780) for 4 h (Huh7 and Hep3B cells) and 6 h (for Hepa1‐6 cells). After that, Hoechst 33342 (5 µg mL^−1^, 10 min) and Mito‐Tracker Green (40 min) were utilized to detect colocalizes with mitochondria via a confocal laser scanning microscope (CLSM, Leica).

### Detection of Intracellular ROS upon Irradiation

A DCFH‐DA kit was used to measure the formation of intracellular ROS. Hep3B, Hepa1‐6, and Huh7 cells were planted into 6‐well or 24‐well plates for 24 h. Then, these cells were treated separately with KET, IR780, P780, KET/P780 NPs or CS@KET/P780 NPs), following a 20 min cultivation with DCFH‐DA (1.0 m) at 37 °C in accordance with the kit's instructions. After washing twice with PBS, the qualitative and quantitative analyses of intracellular ROS formation were evaluated using an inverted fluorescence microscope and flow cytometry.

### ATP Release Assay

The ATP Assay Kit was used to measure the amount of ATP in cells. Cells were cultured for 24 h using six‐well dishes. Following intervention, one technique involved collecting the cell supernatant for further analysis, and the other involved lysing the cells. The cells were lysed and then centrifuged at 12 000 *g* for 5 min at 4 °C to separate the supernatant for further analysis. Following that, the manufacturer's instructions were followed, the 96‐well fluorescence microplate reader was used to the assess fluorescence intensity of ATP.

### Detection of Mitochondrial Membrane Potential

For the detection of early stage apoptosis, the mitochondrial membrane potential was assessed using a JC‐1 assay kit. Primitively, cells were seeded at a density of 5 × 10^4^ cells per well in six‐well plates, and they were incubated at 37 °C for an overnight duration. Using normal saline as a control, the cells were treated with KET, IR780, P780, KET/P780 NPs, and CS@KET/P780 NPs. The cells were treated with normal saline as control group. After 4 h or 6 h (for Hepa1‐6 cells), the cells were reintroduced with new media and treated with laser and then incubated for another 2 h. The mitochondrial potential (ΔΨ*m*) was detected by flow cytometry and fluorescence microscopy as instructed by the manufacturer.

### Detection and Analysis of Apoptosis

Hep3B, Hepa1‐6, and Huh7 cells (5 × 10^4^ cells per well) were plated in six‐well plates and incubated for 24 h. The cells were then subjected to the indicated concentrations of KET, IR780, P780, KET/P780 NPs, and CS@KET/P780 NPs for 4 or 6 h (for Hepa1‐6 cells). The cells were then swapped out for fresh medium and incubated for a further 2 h before being subjected to 660 laser irradiation at 660 nm (for P780) or 808 nm (for IR780) for 30 s (1.0 W cm^−2^). Whereafter, the cells from each well were digested by enzyme, centrifuged, and collected into flow tubes. An appropriate amount of binding buffer was added to each tube to make a single‐cell suspension. Annexin V‐FITC (5 µL) and PI (10 µL) were added into each tube.^[^ ^33^ ^]^ Apoptosis was identified by flow cytometry following a 15 min incubation at room temperature.

### Western Blotting

The cells (5 × 10^4^ cells per well) were plated into six‐well dishes for 24 h incubation and remedied as indicated for 4 h (for Hep3B and Huh7 cells) or 6 h (for Hepa1‐6 cells). As indicated, the cells were followed by an additional 2 h incubation after laser irradiation. The ultrasonic cell disruptor (SCIENTZ‐IID) was then used to lyse the cells using lysis solution while they were chilled. Subsequently, the samples were centrifuged for 10 min at 14 000 rpm and 4 °C. Protein quantification was performed using Coomassie Brilliant Blue.^[^ ^34^ ^]^ Immunoblotting with the relevant antibodies was used to examine the samples.

### Immunofluorescence Assay

Hep3B, Hepa1‐6, and Huh7 cells were incubated on sterilized cover slips in 24‐well plates for 24 h. After the indicated treatment, cells were permeabilized with 0.4% Triton X‐100 and 5% BSA for 1.5 h before being fixed with 4% paraformaldehyde for 30 min. Following a 15 h incubation at 4 °C with the appropriate primary antibodies, a 2 h incubation with Alexa Fluor secondary antibodies was performed at room temperature. After being labeled with DAPI for 8 min, cell nuclei were ten times rinsed in PBS. Images were visualized via a confocal laser scanning microscope (Nikon, Japan).

### Secretion of Prostaglandin E2 (PGE2), Tumor Necrosis Factor (TNF‐α) and Interleukin‐2 (IL‐2)

PGE2, TNF‐α, and IL‐2 of cell supernatants and mouse serum were tested for using ELISA kits (Lianke Biological Co., LTD) following the directions provided by the manufacturer.

### Animals Models

BALB/c nude mice (≈ 20 g), C57BL/6 mice (≈ 20 g) and NSG mice were bought from Chengdu Yaokang Biotechnology Co, Ltd. All in vivo studies were carried out in accordance with the “Guidelines for Animal Experimentation” regulations and were given the go‐ahead by Sichuan University's Treatment Committee. Hep3B cells or Hepa1‐6 cells were subcutaneously injected into each mouse's hindquarter area to create tumors. After the solid tumors reached 80–100 mm^3^ (for Hep3B) or 50–70 mm^3^ (for Hepa1‐6), the mice were split into four groups at random and utilized in the next tests.

### Fluorescence Imaging and Biodistribution Analysis In Vivo/Ex Vivo

When the subcutaneous tumors reached 100 mm^3^ or 80 mm^3^, the tumor‐bearing BALB/c mice and C57BL/6 mice received intravenous injections with free IR780, KET/P780 NPs, CS@KET/P780 NPs via tail vein (3 mice per group, the dosage was equivalent to IR780 of 2 mg kg^−1^). Different points in time (2, 4, 6, 8, 10, 12, and 24 h), fluorescent images and biodistribution were taken with the animal imaging apparatus. The mice's organs were taken out 24 h after injection for *ex vivo* fluorescence imaging.

### In Vivo Thermal Imaging with Infrared

When the subcutaneous tumors reached 80–100 mm^3^ (for BALB/c mice) or 50–70 mm^3^ (for C57BL/6 mice) 100 µL of CS@KET/P780 NPs, KET/P780 NPs and normal saline were injected into tumor‐bearing mice via the tail vein. After 6 h (for BALB/c mice) or 8 h (for C57BL/6 mice), the tumor sites were directly irradiated via 808 nm laser or 660 nm laser (1.0 W cm^−2^) for 3 min. Infrared images were taken using a thermal imaging infrared camera during the irradiation at 1 min intervals.

### In Vivo Therapeutic Efficacy and Biosafety Analysis

The subcutaneous tumor models were created by injection of Hep3B or Hepa1‐6 cells as described above. When the tumor volume achieved to 80–100 mm^3^ or 50–70 mm^3^, the tumor‐bearing mice were divided into 4 groups and injected with 100 µL of normal saline, KET, KET/P780 NPs, or CS@KET/P780 NPs with comparable KET and P780 of 2 mg kg^−1^, respectively (*n* = 5). The other groups, excluding the NS group, were exposed to near‐infrared laser radiation at intervals of 6 h (BALB/c mice) or 8 h (C57BL/6 mice) following each dosage (3 min, 1.0 W cm^−2^). Every 2 d, the intravenous doses were given. Daily records were kept of the tumor volume and body weight. On day 11, the mice were then put to death, their organs were all removed, and the tumors were taken for hematoxylin‐eosin (H&E) and immunohistochemistry analysis.^[^ ^35^ ^]^ The collected Eyeball blood was analyzed for ALT, AST, CRE, and UREA levels.^[^ ^36^ ^]^ To calculate the tumor volume, the formula below was employed: Tumor volume (mm^3^) = *A*×*B* ^2^×0.5. (*A* was the longest tumor diameter, *B* was the shortest tumor diameter) Based on the following equation, the inhibition ratio of tumor‐bearing mice was determined:

(9) $$\mathrm{Tumor}\;\mathrm{inhibition}\;\mathrm{ratio}\;\;\left(\%\right)=\frac{W_{N}-W_{s}}{w_{N}}\times 100\%$$

where *W*_N_ was the average tumor weight of normal saline group, while *W*_s_ was the average tumor weight of the treated group.

### Immune Response Detection In Vivo

The spleens and tumors were collected for staining. Next, the single‐cell suspensions of tumors and spleen were also obtained, and the fluorochrome‐conjugated anti‐mouse antibodies (as shown in **Table** **1**) were used for cell surface tags via flow cytometry.

**Table 1:** The markers of different immune cells and associated antibodies.

Immune cells	Markers	Antibodies
CD4^+^ T cell	CD3^+^ CD4^+^	FITC anti‐mouse CD3, PE anti‐mouse CD4
CD8+ T cell	CD3^+^ CD8^+^	APC anti‐mouse CD8a, FITC anti‐mouse CD3
Treg cells	CD4^+^ CD25^+^ FOXP3^+^	APC anti‐mouse CD4, PE anti‐mouse FOXP3, FITC anti‐mouse CD25
DCs	CD80^+^ CD86^+^ CD11c^+^	FITC anti‐mouse CD80, PE anti‐mouse CD11c, PerCP anti‐mouse CD86
M1 macrophage	CD11b^+^ iNOS^+^	APC anti‐mouse CD11b, PE anti‐Nos2(iNOS)
M2 macrophage	CD11b^+^ CD206^+^	APC anti‐mouse CD11b, PerCP anti‐mouse CD206

### Statistical Analysis

The Prism 8.0 program (GraphPad Software, Inc., La Jolla, CA) was used to analyze all the data, which were presented as mean standard deviation (SD) from at least three distinct trials. A one‐way ANOVA was used to determine whether there is a significant difference between three or more samples of a single variable, a two‐way ANOVA was used to determine whether a difference is statistically significant between three or more samples based on two factors, and the Student's t‐test was employed to determine whether a difference between two samples in a single factor was statistically significant. A statistical difference was deemed to exist when *P* < 0.05 was recorded (*P < 0.05, **P < 0.01, ***P < 0.001).

### Ethics Approval and Consent to Participate

The entire animal testing complied with Sichuan University's institutional guidelines for the use and care of animals.

## Conflict of Interest

The authors declare no conflict of interest.

## Supporting information

Supporting Information

## Data Availability

The data that support the findings of this study are available from the corresponding author upon reasonable request.
