# Supplementary material for: Mitochondrial‐Targeted CS@KET/P780 Nanoplatform for Site‐Specific Delivery and High‐Efficiency Cancer Immunotherapy in Hepatocellular Carcinoma
Source: Adv Sci (Weinh). 2024 Feb 2;11(14):2308027. doi: 10.1002/advs.202308027 (PMC11005749; doi:10.1002/advs.202308027)
Supplement: Supplementary file 1 — Supporting Information [file ADVS-11-2308027-s001.pdf]

ADVANCED SCIENCE
Open Access

Supporting Information

for *Adv. Sci.*, DOI 10.1002/advs.202308027

Mitochondrial-Targeted CS@KET/P780 Nanoplatform for Site-Specific Delivery and High-Efficiency Cancer Immunotherapy in Hepatocellular Carcinoma

Shanshan Liu, Hailong Tian, Hui Ming, Tingting Zhang, Yajie Gao, Ruolan Liu, Lihua Chen, Chen Yang, Edouard C. Nice, Canhua Huang, Jinku Bao, Wei Gao* and Zheng Shi**

Supporting Information

Mitochondrial-Targeted CS@KET/P780 Nanoplatform for Site-Specific Delivery and High-Efficiency Cancer Immunotherapy in Hepatocellular Carcinoma

Shanshan Liu[a,b,1], Hailong Tian[c,1], Hui Ming[c,1] Tingting Zhang[c], Yajie Gao[d], Ruolan Liu[e], Lihua Chen[e], Chen Yang[e], Edouard C. Nice[f], Canhua Huang[c], Jinku Bao[g *], Wei Gao[h *], Zheng Shi[a,b*]

[a]*Clinical Medical College, Affiliated Hospital of Chengdu University, Chengdu University, Chengdu, 610106, China.*

[b]*Department of Clinical Pharmacy, School of Pharmacy, Zunyi Medical University, Zunyi, 563006, China.*

[c]*State Key Laboratory of Biotherapy and Cancer Center, West China Hospital, and West China School of Basic Medical Sciences & Forensic Medicine, Sichuan University, Collaborative Innovation Center for Biotherapy, Chengdu, 610041, China.*

[d]*The First Affiliated Hospital of Ningbo University, Ningbo, 315020, China.*

[e]*School of Basic Medical Sciences, Chengdu University of Traditional Chinese Medicine, Chengdu, 611137, China.*

[f]*Department of Biochemistry and Molecular Biology, Monash University, Clayton, VIC, 3800, Australia.*

[g]*College of Life Sciences, Sichuan University, Chengdu, 610064, China.*

[h]*Clinical Genetics Laboratory, Affiliated Hospital & Clinical Medical College of Chengdu University, Chengdu, 610081, China.*

[1]These authors contributed equally to this work.

*Corresponding authors.

E-mail: baojinku@scu.edu.cn, gaowei@cdu.edu.cn, shizheng@cdu.edu.cn

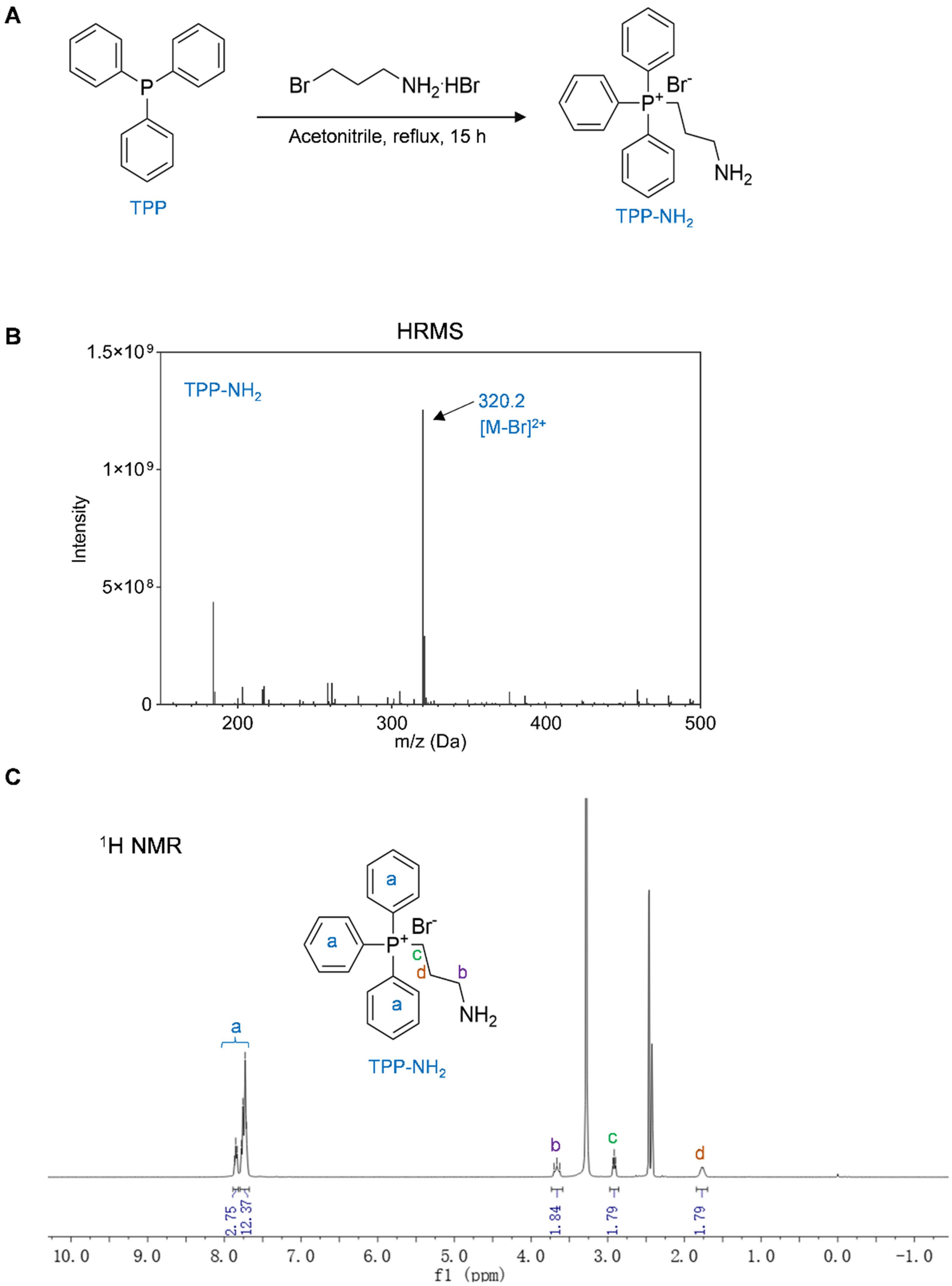

Figure S1. Reagent synthesis of TPP-NH_2. (A) Synthetic route of TPP-NH_2. (B) ^{1}H NMR spectrum of TPP-NH_2 in DMSO-d_6. (C) HR-MS of TPP-NH_2.

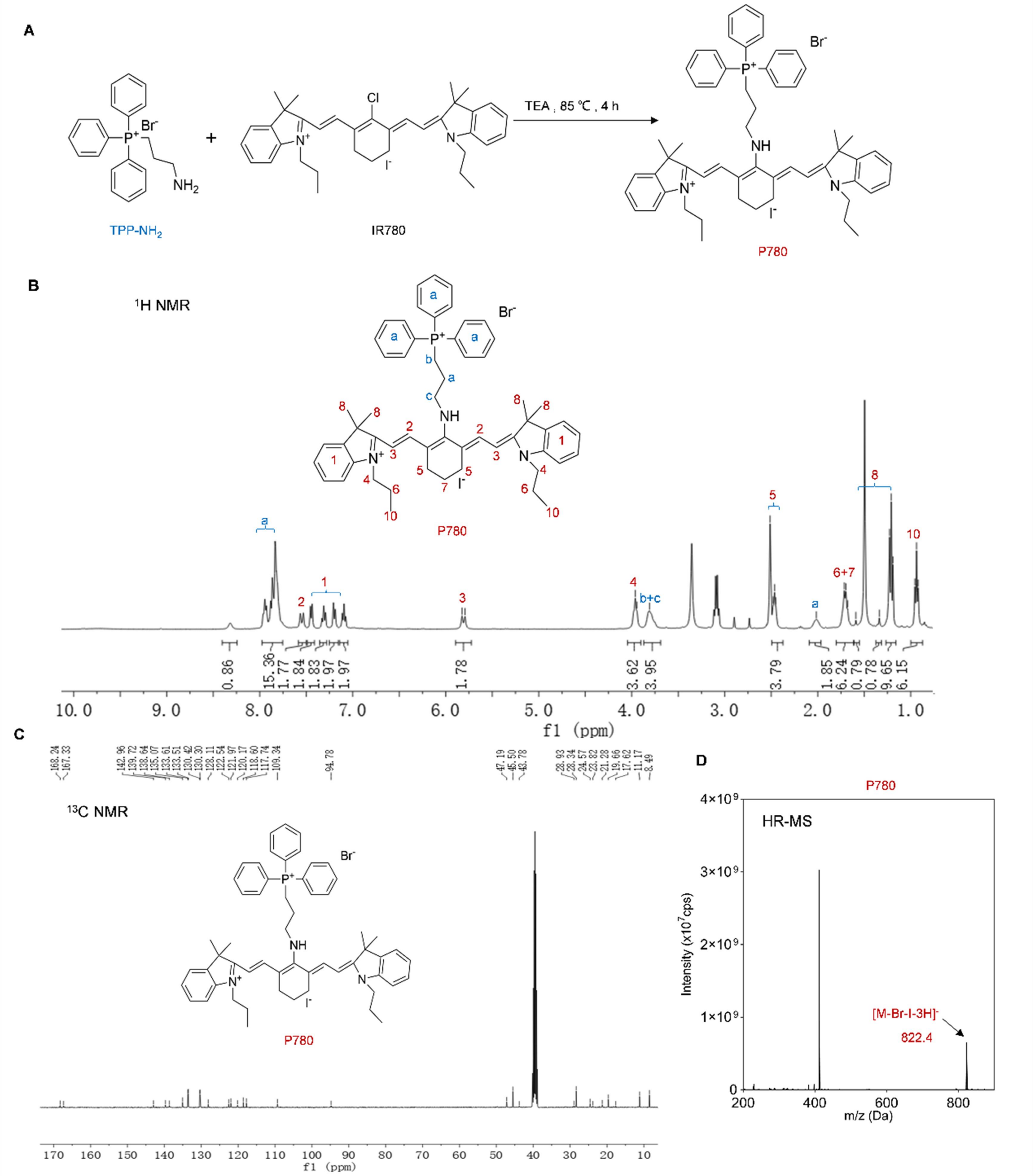

Figure S2. Reagent synthesis of P780. (A) Synthetic route of P780 conjugate. (B) ^{1}H NMR spectrum of P780 in DMSO-d_6. (C) ^{13}C NMR spectrum of P780 in DMSO-d_6. (D) HR-MS of P780.

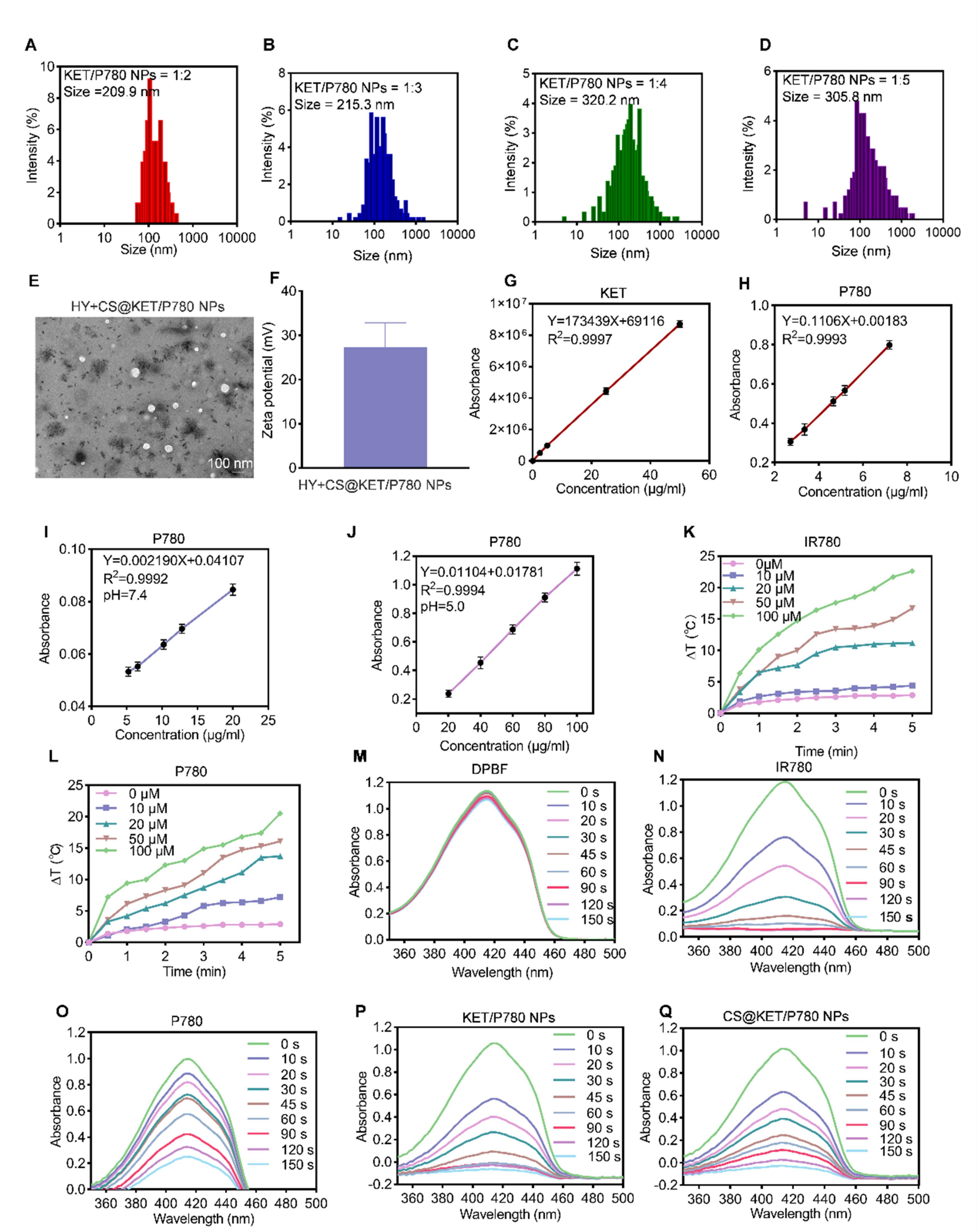

Figure S3. Preparation and characterization of CS@KET/P780 NPs. (The following experimental conditions are: 808 nm for IR780, 660 nm for P780, KET/P780 NPs and CS@KET/P780 NPs; P = 1.0 W cm^{-2}, irradiation time = 30 s; C_{KET} = 4.5 μM, C_{P780} = 2.5 μM). (A-D) Size distribution of KET/P780 NPs at different mass ratios of KET and P780. (E) TEM image of the CS@KET/P780 NPs after cleavage. (F) Zeta potential of the CS@KET/P780 NPs after cleavage. (G) Standard curve of KET was established by HPLC (n = 3). (H) Standard curve of P780 (n = 3). (I-J) Standard

curves of P780 at pH values of 7.4 and 5.0 (n = 3). (K-L) The photothermal efficiency of IR780 and P780 distributed in water with indicated concentrations. (M-Q) Levels of DPBF that remain after laser irradiation in the given groupings (150 s, 1.0 W cm^{-2}).

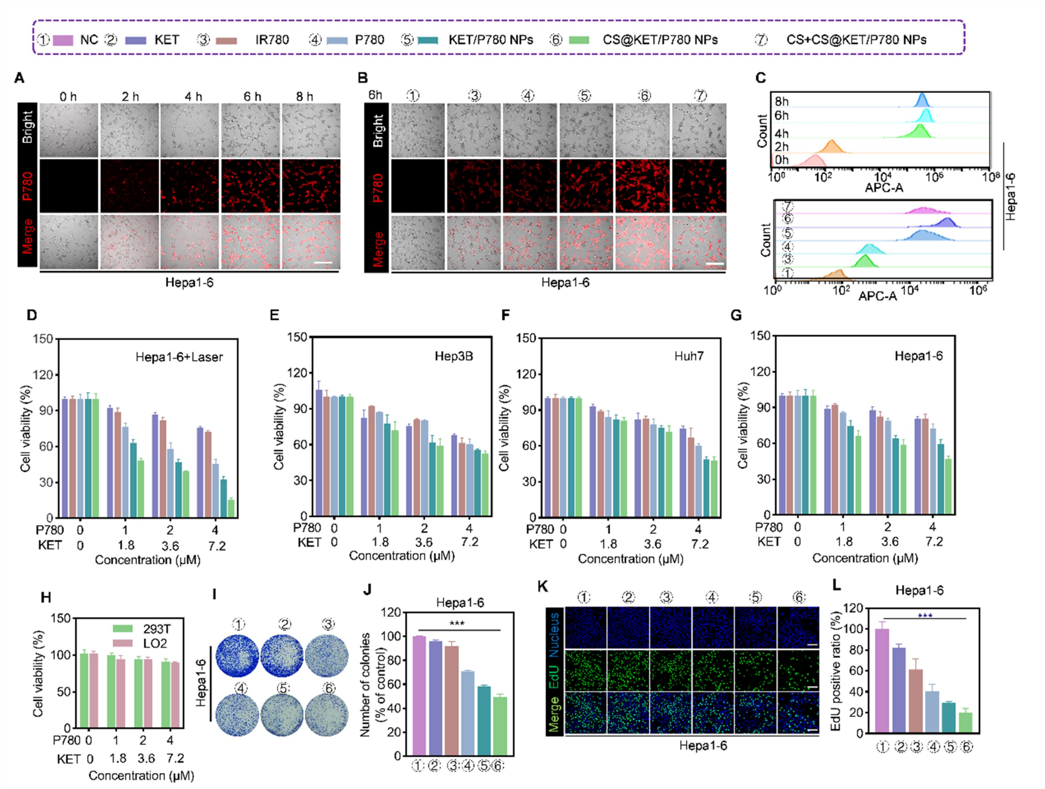

Figure S4. The cellular uptake and cytotoxicity of CS@KET/P780 NPs *in vitro*. (The following experimental conditions are: 808 nm for IR780, 660 nm for P780, KET/P780 NPs and CS@KET/P780 NPs; P = 1.0 W cm^{-2}, irradiation time = 30 s; C_{KET} = 4.5 μM, C_{P780} = 2.5 μM). (A) Images captured by fluorescence microscopy show the cellular uptake of CS@KET/P780 NPs in Hepa1-6 cells at various intervals. Scale bar: 50 μm. (B) Fluorescence microscopy pictures of the cellular uptake of IR780, P780, KET/P780 NPs, CS@KET/P780 NPs, and CS+CS@KET/P780 NPs (cells were treated with CS for half an hour beforehand) in Hepa1-6 cells following 4-hour incubation. Scale bar: 50 μm. (C) Flow cytometry results of corresponding cellular uptake in Hepa1-6 cells. (D) Viability of Hepa1-6 cells treated with KET, IR780, P780, KET/P780 NPs, and CS@KET/P780 NPs after NIR laser irradiation.

(E-G) Viability of Hep3B, Huh7, and Hepa1-6 cells treated with CS@KET/P780 NPs, KET/P780 NPs, P780, IR780 and KET without laser irradiation. (H) The viability of LO2 and 293 T cells following treatment with CS@KET/P780 NPs at different concentrations. (I-J) Representative images for colony development and quantitative analysis of Hepa1-6 cells under different treatments. (K-L) EdU labeling test quantitative analysis and fluorescence microscopy in Hepa1-6 cells with various treatments. Scale bar: 50 μm. (****P* < 0.001, one-way ANOVA).

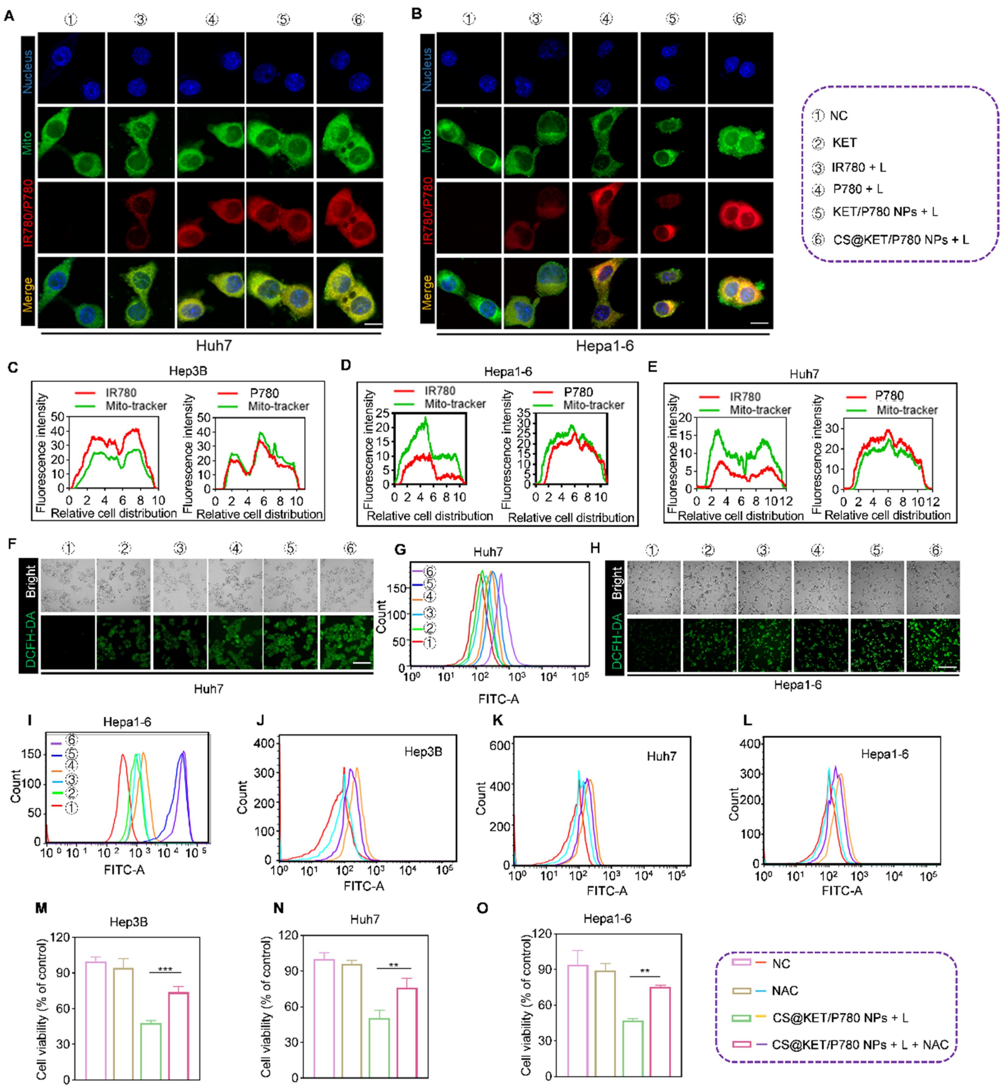

Figure S5. CS@KET/P780 NPs cause ROS buildup and mitochondrial dysfunction in liver cancer cells (808 nm for IR780, 660 nm for P780, KET/P780 NPs and

CS@KET/P780 NPs; P = 1.0 W cm^{-2}, irradiation time = 30 s; C_{KET} = 4.5 μM, C_{P780} = 2.5 μM). (A-B) LSCM images to display subcellular localization of P780 or IR780 in Huh7 and Hepa1-6 cells under different therapies. Scale bar: 10 μm. (C-E) Pearson's correlation coefficient analysis of the co-location with mitochondria in HCC cells under different treatments. (F, H) Fluorescence images and (G, I) intracellular ROS levels of Huh7 and Hepa1-6 cells examined using flow cytometry DCFH-DA probe. Scale bar: 100 μm. (J-L) Analysis of intracellular ROS production of NAC-treated Hep3B, Huh7, and Hepa1-6 cells using flow cytometry. (M-O) Hep3B, Huh7, and Hepa1-6 cells viability of certain populations with or without NAC (10 μM) treatment. (***P* < 0.01; ****P* < 0.001, one-way ANOVA).

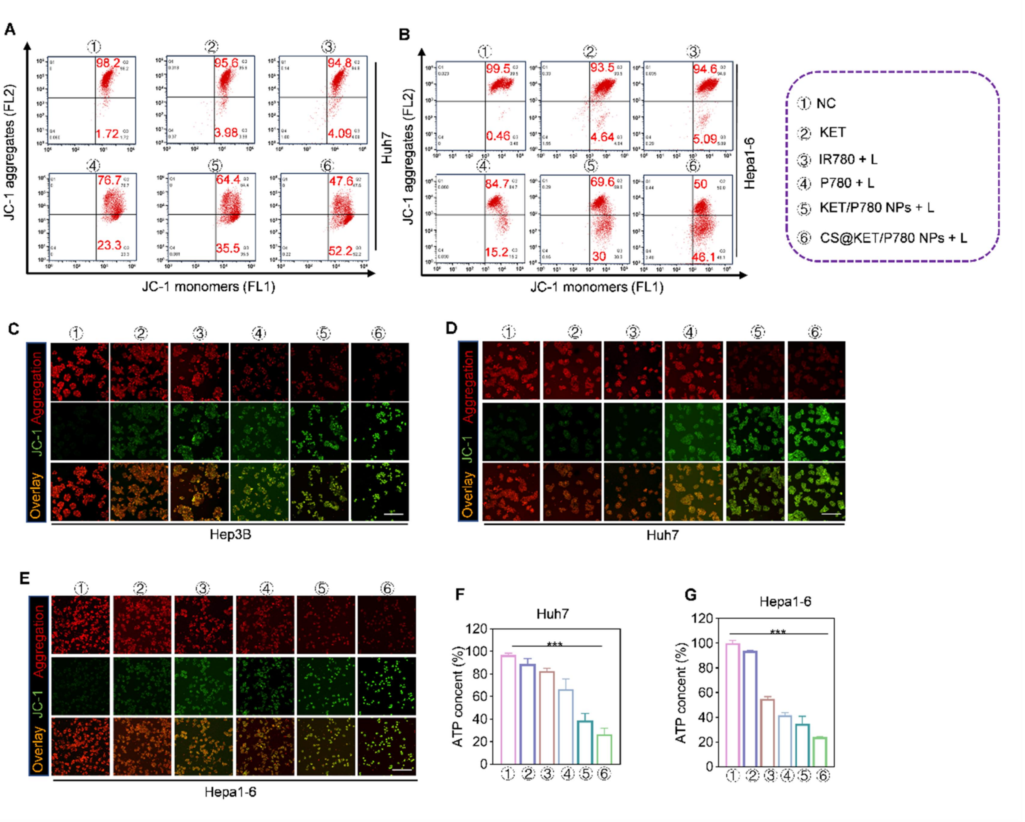

Figure S6. CS@KET/P780 NPs induces mitochondrial dysfunction in HCC cells (808 nm for IR780, 660 nm for P780, KET/P780 NPs and CS@KET/P780 NPs; P = 1.0 W cm^{-2}, irradiation time = 30 s; C_{KET} = 4.5 μM, C_{P780} = 2.5 μM). (A-B) Flow cytometry investigation for potential of the mitochondrial membrane of Huh7 and Hepa1-6 cells following different treatments. (C-E) Fluorescence images for

mitochondrial membrane potential of Hep3B, Huh7, and Hepa1-6 cells determined by JC-1 assay. Scale bar: 100 μm. (F-G) ATP content in Huh7 and Hepa1-6 cells following different treatments. (***$P < 0.001$, one-way ANOVA).

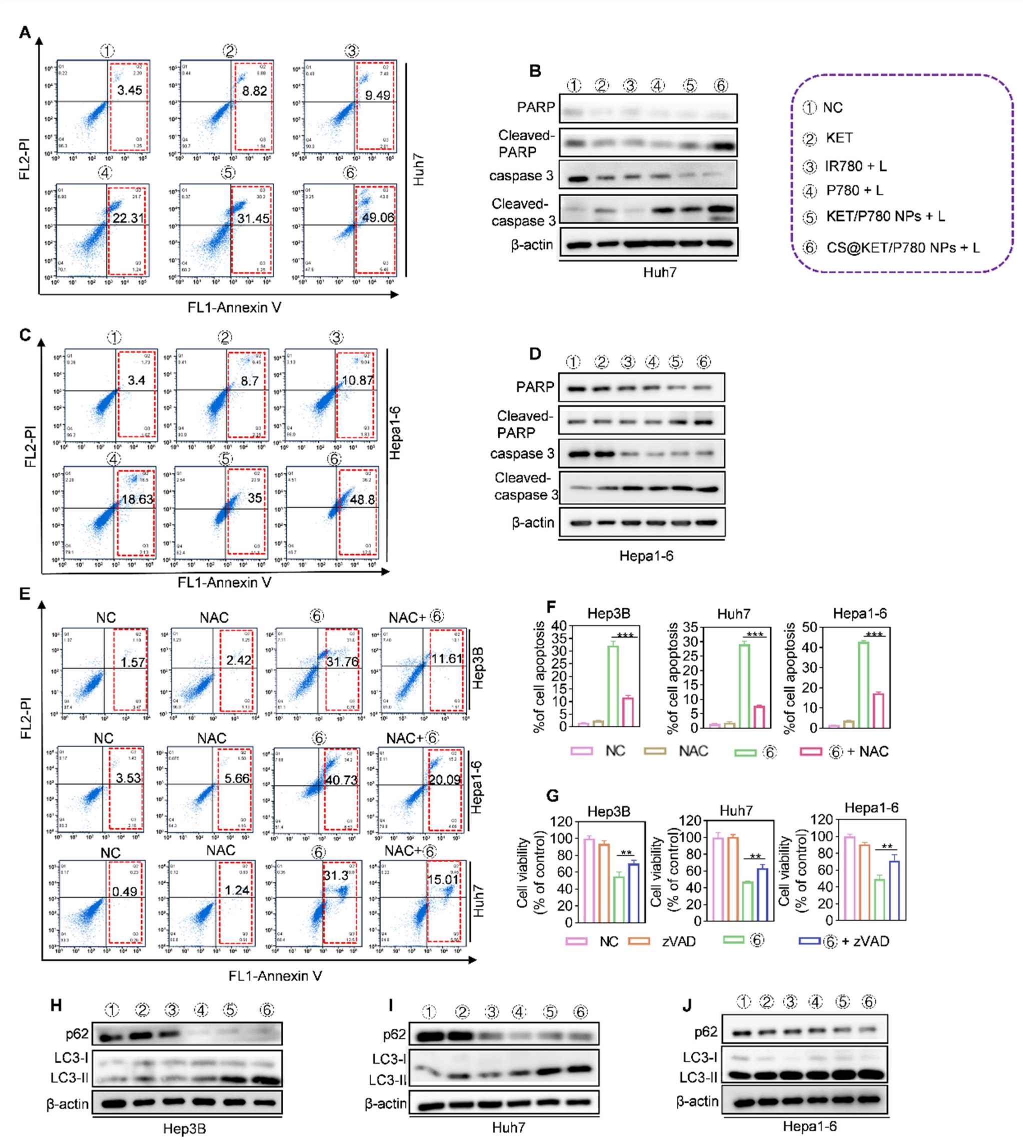

Figure S7. CS@KET/P780 NPs evoke apoptosis through ROS accumulation in liver cancer cells (808 nm for IR780, 660 nm for P780, KET/P780 NPs and CS@KET/P780 NPs; P = 1.0 W cm^{-2}, irradiation time = 30 s; C_{KET} = 4.5 μM, C_{P780} = 2.5 μM). (A, C) Results of apoptosis in Huh7 and Hepa1-6 cells via flow cytometry after various treatments. (B-D) Analysis of apoptotic markers using Western blot for Huh7 and Hepa1-6 cells after various treatments. (E-F) Flow cytometry results and

quantification of apoptotic cell ratio of apoptosis in Huh7, Hep3B, and Hepa1-6 cells treated with NC, NAC, CS@KET/P780 NPs and CS@KET/P780 NPs+NAC. (G) Cell viability of particular cell populations in Hep3B, Huh7, and Hepa1-6 cells with or without ZVAD therapy. (H-J) Western blot analysis of autophagic markers for Hep3B, Huh7, and Hepa1-6 cells treated with KET, IR780, P780, KET/P780 NPs and CS@KET/P780 NPs. (**$P < 0.01$, ***$P < 0.001$, one-way ANOVA).

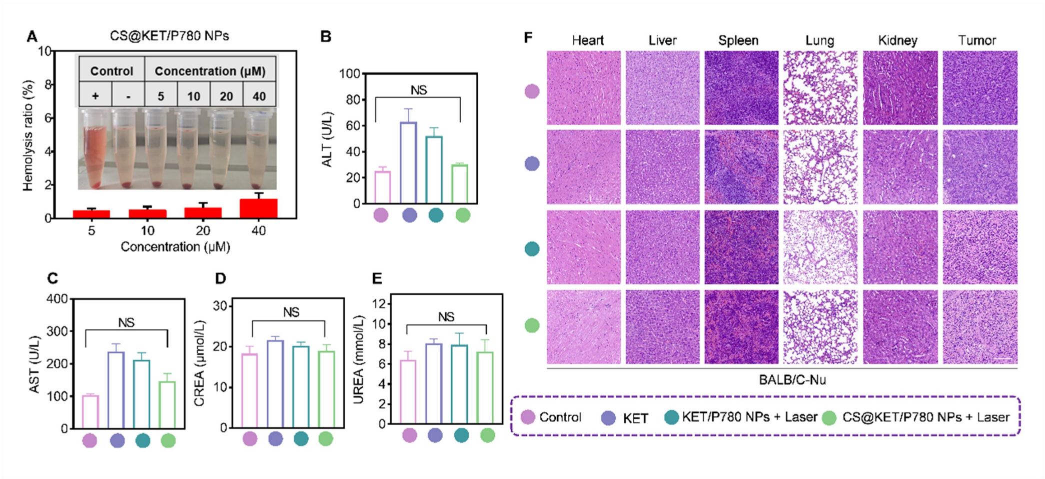

Figure S8. *In vivo* biosafety assessment of CS@KET/P780 NPs. (A) Hemolysis rate and photographs of CS@KET/P780 NPs at different concentrations. (B-E) Analysis of the serum biochemistry indicators (ALT (B); AST (C); CREA (D); UREA (E)) after various treatments. (F) H&E staining of the major organs and tumor tissue in a variety of therapeutic groups (Scale bars: 50 μm, NS, not significant).

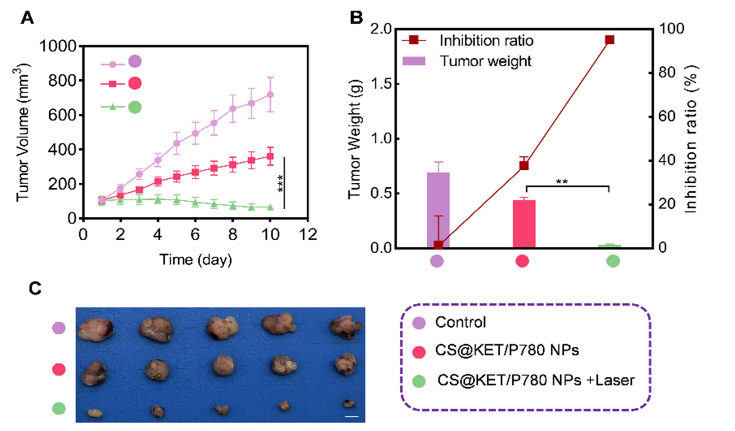

Figure S9. *In vivo* anti-liver cancer performance of CS@KET/P780 NPs in C57BL/6

mice. The mice were treated with normal saline, CS@KET/P780 NPs without laser irradiation, or CS@KET/P780 NPs with laser irradiation (λ = 660 nm, P = 1.0 W cm^{-2}; irradiation time = 3 min). (A) Tumor volume curves of different groups (n = 5). (B) The weight of individual tumors and the inhibition ratio. (C) Photographs of the dissected tumors of different groups (n = 5). (***P* < 0.01; ****P* < 0.001, one-way ANOVA).
